# Multi‐Color Flexible Electrochromic Device for Smart Anti‐Counterfeiting

**DOI:** 10.1002/anie.6863859

**Published:** 2026-04-13

**Authors:** Feifei Zhao, Bingkun Huang, Kaili Gong, Bin Wang, Anni Sun, Yilin Liu, Wu Zhang, Jingwei Chen, Haizeng Li, William W. Yu

**Affiliations:** ^1^ School of Chemistry and Chemical Engineering, Ministry of Education Key Laboratory of Special Functional Aggregated Materials, Shandong Key Laboratory of Advanced Organosilicon Materials and Technologies Shandong University Jinan China; ^2^ Shandong Provincial Key Laboratory for Science of Material Creation and Energy Conversion, Science Center for Material Creation and Energy Conversion Shandong University Qingdao China; ^3^ Institute of Frontier and Interdisciplinary Science Shandong University Qingdao China; ^4^ Ultrafast Optics and Nanophotonics Laboratory, Department of Electrical and Computer Engineering University of Alberta Edmonton Alberta Canada; ^5^ School of Materials Science and Engineering Ocean University of China Qingdao China

**Keywords:** electrochromism, information encryption, multicolor display, Prussian blue analogue, zinc anode‐based electrochromic device

## Abstract

Information security is critically important. We propose a multilevel electrochromic display design for dynamic information encryption, enabled by Prussian blue, zinc, and potassium nickel hexacyanoferrate. The as‐fabricated electrochromic devices offer a two‐dimensional CIE color space modulation with four distinct states from transparent to blue, green, and yellow. The devices facilitate precise, localized, and dynamic modulation of electrochromism via an elaborately designed and independently addressable pattern configuration, enabling advanced encryption and identity authentication with enhanced adaptability. This approach achieves superior security through multi‐stage authentication, real‐time color modulation, and adjustable smart encryption levels tailored to different specific requirements. Our work envisions a new generation of flexible electrochromic devices that elevate both display performance and information security.

## Graphical abstract

A versatile information encryption‐decryption platform using Zn anode‐based electrochromic devices enables multicolor, on‐demand, and multiple encryption modes, breaking through the limitations of conventional static displays that rely on static, pre‐designed patterns.

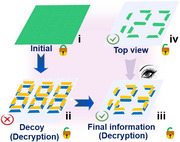

## 1 Introduction

Information encryption has become increasingly vital in today's hyper‐connected society [1, 2]. In information security frameworks, displays serve as the visual medium through which data are concealed, verified, or read. Among the various encryption technologies, luminescent materials including fluorescent [3] and phosphorescent [4, 5] compounds have shown great promise due to their high brightness, rapid responsiveness, and ease of visualization. However, achieving both high information security and efficient data extraction using luminescent systems remains a significant challenge [6, 7]. These materials often exhibit time‐dependent behavior [4] and limited operational lifespans [3], restricting their practical applicability. While electroluminescent technologies (e.g., organic light‐emitting diodes and quantum‐dot light‐emitting diodes) offer immediate light emission upon electrical stimulation and avoid time‐dependent activation, they rely on a sustained power supply, and prolonged operation degrades the materials [8]. Stimulus‐responsive color‐changing materials, such as thermochromic [9] and photochromic [10] compounds, offer dynamic encryption capabilities through color modulation. However, their practical deployment is further limited by high‐temperature requirements [9], inadequate fatigue resistance [10], and environmental sensitivity, which compromise long‐term stability and reliability in high‐frequency operation.

Electrochromism modulates optical properties along with reversible ion insertion and extraction, enabling stable visual states with minimal energy consumption [11, 12, 13, 14, 15, 16]. Studies have demonstrated that electrochromic devices (ECDs) can effectively manipulate optical characteristics to achieve secure data encryption [17]. Integrating electrochromic displays into encryption and decryption systems provides an innovative approach for enhancing data protection, making it a promising solution in information security [18].

Electrochromic displays offer low‐power color switching while maintaining visual states without continuous energy input [19, 20]. However, conventional electrochromic systems rely on the intrinsic properties of their materials to generate color, resulting in a limited color gamut and insufficient transition dynamics [21, 22, 23, 24, 25, 26, 27]. Moreover, rigid electrochromic displays suffer from inflexible substrates and a lack of region‐specific modulation, hindering their integration into emerging platforms like wearable electronics, smart packaging, and adaptive interfaces. To meet the growing demand for secure, customizable, and interactive systems, it is essential to develop flexible, multi‐mode, and multi‐color ECDs featuring independently and on‐demand controllable functionalities for advanced encryption and decryption applications.

Zinc anode‐based electrochromic devices (ZECDs) offer intrinsic advantages for multicolor displays, including a simplified architecture and the independent operation of each electrochromic layer.[12, 14, 16, 28, 29] ZECDs can enable two‐dimensional (2D) International Commission on Illumination (CIE) color space modulation by combining different electrochromic materials (such as WO_3_ and V_2_O_5_ [19]). Compared to conventional materials such as WO_3_, Prussian blue (PB) and its analogues (PBAs) offer tunable properties and cost‐effective aqueous synthesis [30, 31]. Their superior optical modulation and vibrant color expression render them promising candidates for next‐generation electrochromic displays.

In this work, Prussian blue (PB)‐zinc‐potassium nickel hexacyanoferrate (NiHCF) zinc‐anode electrochromic devices (ZECDs) achieved a breakthrough 2D CIE color space four‐color modulation (transparent‐blue‐green‐yellow), attributed to the independently tunable nature of the ZECD system and a distinctive color overlay effect. The PB‐Zn‐NiHCF ZECDs with a 5 × 5 cm^2^ area demonstrate outstanding dynamic visible modulation (optical modulation Δ*T* = 53.6% at 632 nm and Δ*T* = 31.5% at 420 nm) and rapid switching times (bleaching time *t*_b _= 15.4 s and coloration time *t*_c _= 9.2 s between green and transparent at 632 nm; *t*_b _= 25.6 s and *t*_c _= 8.0 s for coloration between yellow and transparent at 420 nm). These results indicate that the PB‐Zn‐NiHCF ZECDs demonstrate superior overall performance for further advanced applications. We employed an advanced flexible transparent conductive substrate and designed three independently addressable electrode regions, thus enabling precise, localized color modulation and reversible transitions from transparent to distinct color states such as yellow, blue, and green. Leveraging this technology, we developed a versatile information encryption‐decryption platform featuring multicolor, on‐demand display and multiple encryption modes, breaking through the limitations of conventional static displays that require pre‐designed patterns. This work envisions a new generation of flexible ECDs that not only enhance display performance, but also elevate the standards of information security.

## 2 Results and Discussion

### 2.1 Electrochromic Performance of Zn‐NiHCF ZECD

The yellow NiHCF nanoparticles, with an average size of 20 nm, were synthesized via a straightforward one‐pot method (Figure S1a). X‐ray diffraction (XRD) analysis in Figure S1b confirmed the cubic phase with space group Fm‐3m (225) of the NiHCF (KNi[Fe(CN)_6_]) nanoparticles (Joint Committee on Powder Diffraction Standards No. 51–1897). We subsequently fabricated yellow NiHCF electrodes by spraying stable aqueous dispersions of NiHCF onto ITO/glass, achieving a transmittance of 20.1% at 420 nm (Figure S1c). This facile and scalable technique supports rapid fabrication of large‐area films. The resulting electrodes exhibited a rough and porous morphology with an approximate thickness of 1.5 µm (Figure S1d), which provided an enhanced ion‐buffering capacity and facilitated efficient ion transport, thereby improving electrochromic performance.

Previous studies have demonstrated that K^+^ ions exhibit excellent compatibility with PBAs due to their ability to readily diffuse into the open framework structure of the PBA lattice, enabling highly reversible and rapid intercalation kinetics [29, 32]. Zn^2+^ plays an important role in ZECD for charge balance. Therefore, we formulated a mixed 0.1 M Zn^2+^–0.9 M K^+^ electrolyte for the Zn‐NiHCF electrochemical platform. We investigated the electrochromic performance of the spray‐coated NiHCF electrode using a well‐designed two‐electrode configuration, where zinc foil and the NiHCF electrode served as the anode and cathode, respectively. To address the poor stability of the NiHCF electrode in aqueous Zn^2+^‐K^+^ electrolytes (Figure S2) [33], we employed a polyacrylamide (PAM) hydrogel containing 1 M Zn^2+^‐K^+^ as electrolyte (Figure S3). The flexible PAM hydrogel exhibited high optical transmittance (>85% across 400–800 nm, Figure S3a), outperforming our previously reported PVA‐based photochromic hydrogels (<85% in visible light) [34] and demonstrating excellent potential for transparent electrochromic applications. SEM image (Figure S3b) reveals a uniform porous network with pore diameters ranging from 10 to 55 µm, facilitating efficient ion transport and storage capabilities. Electrochemical alternative current (AC) impedance spectrum (Figure S3c) indicates a high ionic conductivity of 2.4 S·m^−1^ for the PAM hydrogel, further validated by its ability to successfully power an LED (inset in Figure S3c). Moreover, the hydrogel showed outstanding stretchability and twistability (Figure S3d), ensuring compatibility with flexible device architectures. The PAM hydrogel electrolyte also showed a stable electrochemical window, which ensures the long‐term operational reliability of the device (Figure S4).

**FIGURE 1:**
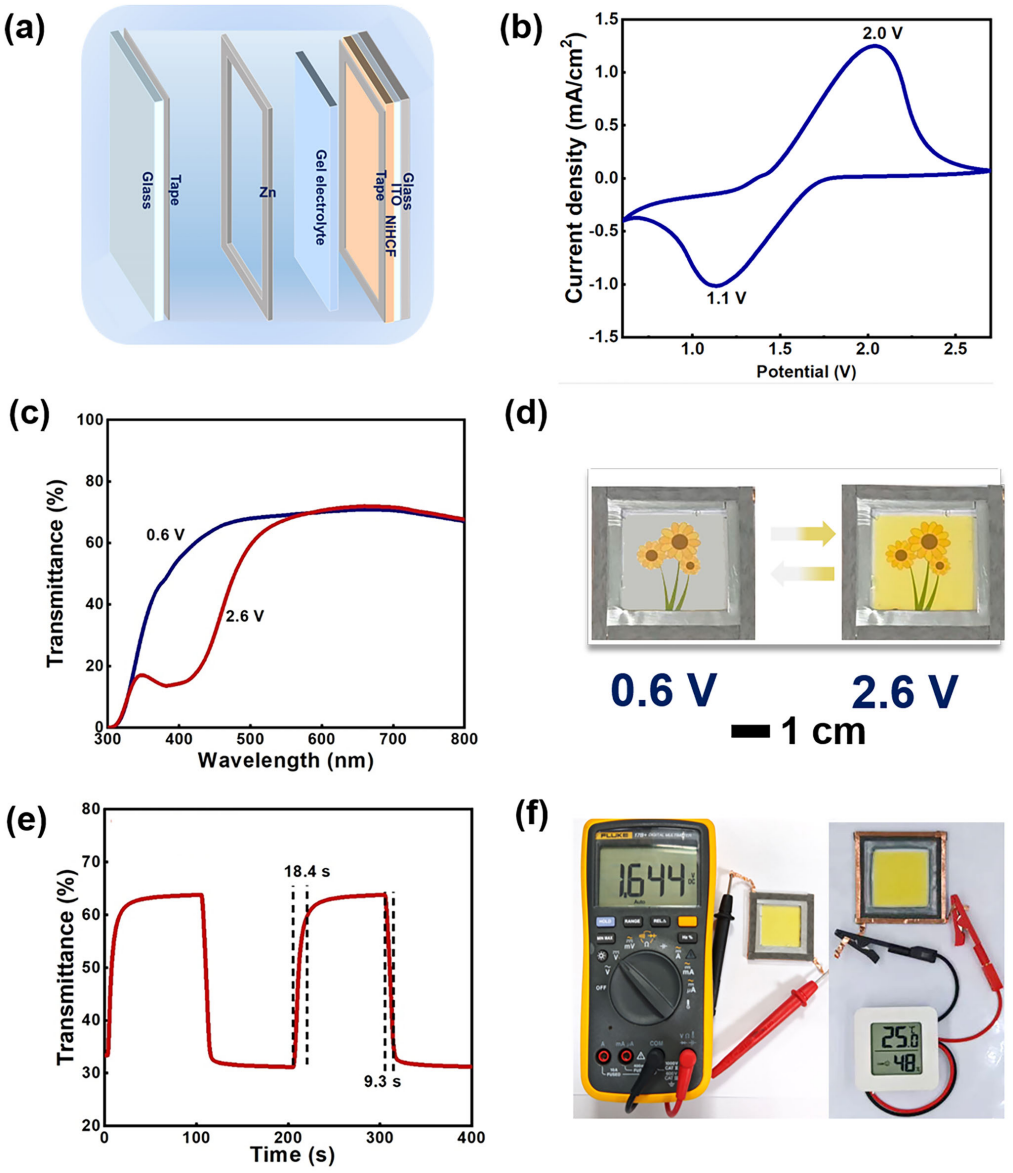
Electrochromic performance of the Zn‐NiHCF ZECD prototype device. (a) Schematic diagram of the device configuration. (b) Cyclic voltammograms of the device in a PAM hydrogel electrolyte system containing 1 M Zn(ClO_4_)_2_‐KCl at a scan rate of 50 mV s^−1^ over a voltage range of 0.6–2.6 V. (c) Optical transmittance spectra and (d) corresponding digital photographs of the device in the bleached and colored states. (e) Real‐time transmittance changes measured at 420 nm in the 0.6–2.6 V window. (f) Digital photographs depicting the device with an OCP of 1.64 V (left), and a temperature‐humidity display powered by the device in colored state (right).

We assembled a Zn‐NiHCF prototype (5 × 5 cm^2^) to evaluate the device performance. It comprises a NiHCF cathode, a Zn anode, and a PAM gel electrolyte, as illustrated in Figure 1a. To improve the film uniformity and electrical conductivity of the NiHCF nanoparticle layer, small amounts of highly conductive materials were incorporated into the aqueous dispersion during film preparation (Figures S4–S6). Compared to pristine NiHCF (Figure S5), NiHCF with poly(3,4‐ethylenedioxythiophene):poly(styrenesulfonate) (PEDOT:PSS) (Figure S6), and NiHCF with 5 µL Mxene (Figure S7), the addition of 10 µL MXene (Figure 1) resulted in significantly improved optical modulation and response times. The redox potential difference between the NiHCF electrode and the zinc foil served as the driving force for the oxidation of Zn and reduction of NiHCF, initiating a spontaneous thermodynamically downhill process resembling the discharging behavior of a secondary battery [12, 16, 32]. During the discharging process, the redox potential difference prompts the oxidation of the Zn anode (Zn→Zn^2+^) and intercalation of K^+^ into the NiHCF electrode, resulting in a self‐bleaching effect. In the charging process, an external voltage drives the deintercalation of K^+^ from the NiHCF electrode and reduction of Zn^2+^ (Zn^2+^→Zn). Figure 1b depicts the cyclic voltammetry (CV) curve of the Zn‐NiHCF prototype device, showcasing a pair of well‐defined redox peaks at 1.1 and 2.0 V, corresponding to the reduction/oxidation processes of low‐spin Fe^II^/Fe^III^via the intercalation/extraction of K^+^ [35].

The device demonstrated a wider potential window of 0.9 V, along with enhanced material stability and electrochemical activity [33, 36, 37]. The Zn‐NiHCF exhibited remarkable optical performance, achieving a high transmittance modulation (Δ*T* = 32.6% at 420 nm, Figure 1c) with distinct bleached (transparent) and colored (yellow) states (Figure 1d). Dynamic optical transmittance measurements reveal rapid color switching kinetics of the Zn‐NiHCF platform between transparent and yellow states (0.6–2.6 V), demonstrating fast response times of 18.4 s (*t*_b_) and 9.3 s (*t*_c_) at 420 nm (Figure 1e), showcasing its superior electrochromic performance. The corresponding coloration efficiency (CE) value of Zn‐NiHCF is about 42.5 cm^2^ C^−1^ (Figure S8). Notably, the Zn‐NiHCF platform exhibits a high built‐in voltage (open‐circuit potential, OCP) of 1.64 V in the yellow state (fully charged state), as shown on the left of Figure 1f. This voltage originated from the potential difference between the NiHCF cathode and the Zn anode [12, 28]. This OCP enables the device to successfully power a temperature‐humidity display for 95 min (right side of Figure 1f), thereby spontaneously achieving a color switching from yellow to transparent. This color‐switching behavior parallels the operational mechanism of a secondary battery [12, 13]. Impressively, the Zn‐NiHCF device maintained 85% of its original optical contrast after 1000 cycles (Figure S9a), demonstrating superior cycling stability compared to the Zn‐NiHCF system utilizing an aqueous electrolyte (37.1% retention; Figure S9b). Notably, even with the gel electrolyte, Zn dendrites were observed on the Zn anode after 1000 cycles, and such dendrite formation is closely associated with the performance decay of the device (Figure S10). The Zn‐NiHCF device's improved cycling stability stems from its optimized gel electrolyte and enhanced interfacial compatibility, which together minimize degradation and preserve electrochromic performance over time [38].

### 2.2 Electrochromic Performance of Zn‐PB ZECDs

While the NiHCF electrode exhibits excellent electrochromic properties, its color gamut can only be modulated within a linear segment scope in the CIE color space between yellow and transparent (Figure S11). To enable a wider color gamut, we integrated conventional PB with the NiHCF electrode to achieve multi‐color displays. We synthesized PB (Fe_4_[Fe(CN)_6_]_3_, Joint Committee on Powder Diffraction Standards No. 01–0239) nanoparticles with an average size of 10 nm, and fabricated a PB electrode via spray coating with a thickness of approximately 208 nm (Figure S12a–c). The resulting blue PB film has a transmittance of 5% at 632 nm (Figure S12d). Subsequently, we constructed a Zn‐PB prototype device (5 × 5 cm^2^) composed of a PB cathode, a Zn anode, and a PAM gel electrolyte (Figure S13). CV measurements show that the Zn^2+^‐K^+^ hybrid electrolyte (Figure S13a) exhibits distinct redox peaks at 0.9 and 1.55 V, along with a significantly larger CV area compared to the single Zn^2+^ electrolyte (Figure S14), indicating enhanced electrochemical activity. The Zn‐PB device demonstrates outstanding optical performance, achieving a high transmittance modulation (Δ*T* = 63% at 632 nm; Figure S13b) and clearly distinguishable transparent and blue states (Figure S13c). Dynamic transmittance measurements exhibit rapid switching kinetics, with *t*_b_ and *t*_c_ of 16.7 and 12.9 s at 632 nm, respectively (Figure S13d), confirming its superior electrochromic performance. Moreover, the Zn‐PB platform delivers exceptional coloration efficiency (CE), reaching 167.2 cm^2^ C^−^^1^ at 632 nm (Figure S13e), surpassing the CE values of current state‐of‐the‐art technologies [29, 39, 40]. Notably, the Zn‐PB device exhibits superior cycle performance and retains 92% of its initial optical contrast after 1000 cycles (Figure S13f). Besides, the device also exhibits a high OCP of 1.34 V in its fully charged (blue) state (Figure S13g), which is sufficient to power an LED (Figure S13h). This capability enables spontaneous color switching from blue to transparent, paralleling the discharge behavior of a secondary battery. Single Zn‐PB shows great electrochromic properties, but the color tunability within a linear CIE color space, thus leading to limited color hues (Figure S15). Combining PB and NiHCF into one device will broaden the color gamut and realize a 2D CIE color space.

### 2.3 Electrochromic Performance of PB‐Zn‐NiHCF ZECDs

**FIGURE 2:**
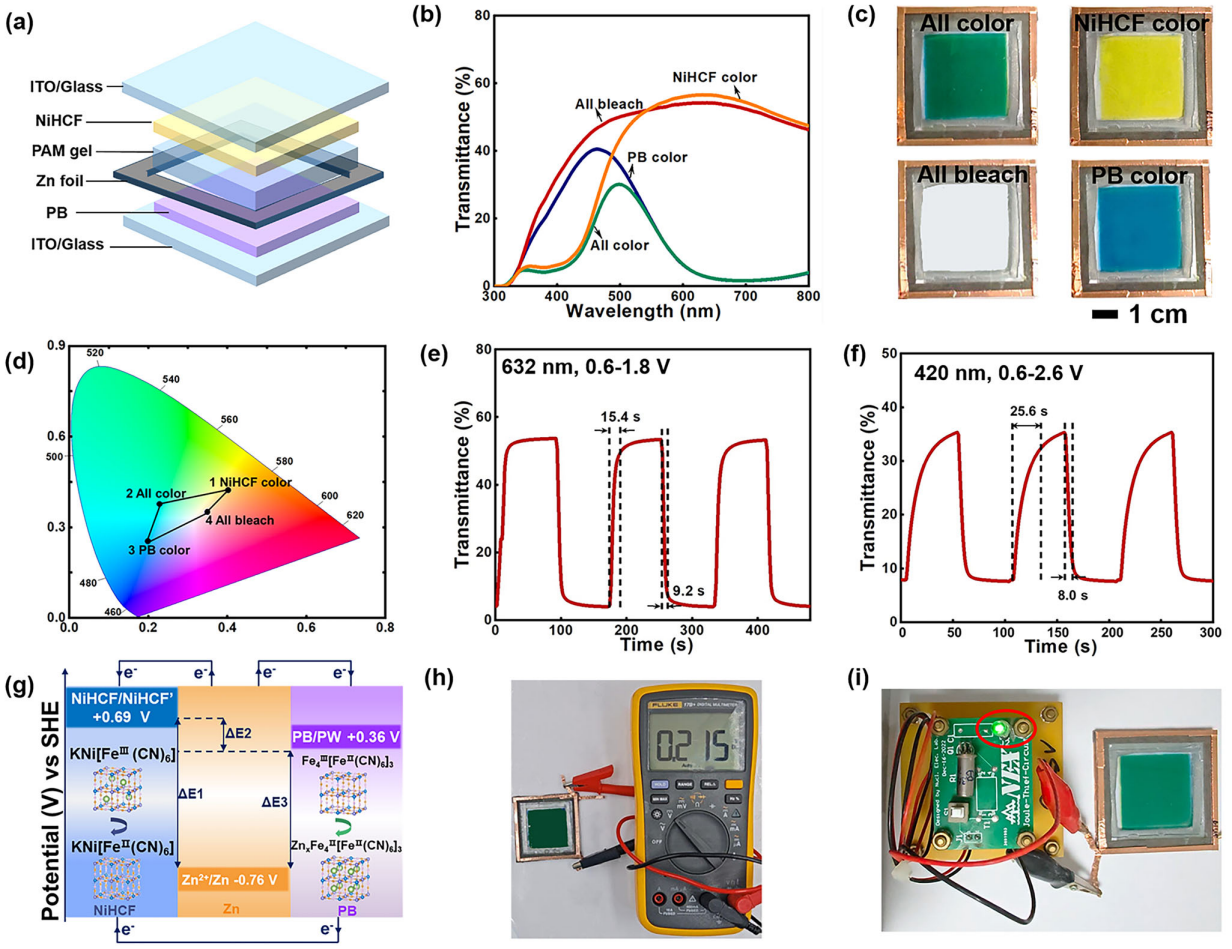
Electrochromic performance of the PB‐Zn‐NiHCF ZECD prototype device. (a) Schematic diagram of the device configuration. (b) Optical transmittance spectra and (c) corresponding digital photographs of the device in the four states: all colored (green), all bleached (transparent), PB color (blue), and NiHCF color (yellow). (d) CIE color coordinates (the four labeled numbers correspond to the four colors in c) of the PB‐Zn‐NiHCF device at different color states. Real‐time transmittance changes measured at (e) 632 nm in the 0.6–1.8 V window and (f) 420 nm in the 0.6–2.6 V window. (g) Energy level transition diagram of Zn, PB, and NiHCF in the K^+^‐Zn^2+^ electrolyte system. NiHCF’: the reduced NiHCF. Digital photographs depicting the device (h) with an OCP of 0.215 V, and (i) an LED powered by the built‐in voltage between PB and NiHCF electrodes in their colored states via a “Joule thief circuit”.

As illustrated in Figure 2a, the PB‐Zn‐NiHCF ZECDs feature a stacked architecture, comprising a PB electrode layer above a NiHCF electrode an electrochromic cathode, with a zinc foil sandwiched between them as an anode. This configuration enables the two electrode layers to operate independently, allowing for dynamic electrochromic activation pathways and a broader range of chromatic responses. The ability to individually control the coloration and bleaching of each layer, in fact, offers precise optical modulation, resulting in distinct transmittance spectra (Figure 2b). As shown in Figure 2c, when the PB electrode layer is fully charged (1.8 V) and the NiHCF electrode layer is bleached (0.6 V), the device displays a blue PB state with transmittance values of 3.1% at 632 nm (colored PB) and 32.8% at 420 nm (bleached NiHCF); conversely, when NiHCF layer is charged (2.6 V) and PB layer is bleached (0.6 V), the device exhibits a yellow NiHCF state with transmittance values of 8.1% at 420 nm (colored NiHCF) and 56.6% at 632 nm (bleached PB); simultaneous activation of both electrode layers (PB at 1.8 V, NiHCF at 2.6 V) results in a green mixed state, showing 6.5% at 420 nm and 3.0% at 632 nm; while full bleaching of both electrode layers (0.6 V each) yields a transparent state with transmittance values of 38.1% at 420 nm and 54.2% at 632 nm. Video S1 shows the rapid and reversible color switching across these four distinct states.

Zn‐NiHCF and Zn‐PB devices exhibit one‐dimensional (1D) color transitions, following linear trajectories in the CIE color space (Figures S11, S15), which restricts their color‐tuning capability to a limited gamut. In contrast, the Zn‐anode‐based electrochromic display leverages a flexible dual‐layer architecture to enable a unique color overlay effect, resulting in two‐dimensional (2D) tunability within the CIE color space. This structure significantly expands the accessible color gamut to an area of 8.97% in the CIE 1931 chromaticity diagram and offers enhanced color control for high‐quality display applications, surpassing the color gamut achieved by current state‑of‑the‑art electrochromic devices [19, 21, 41, 42, 43, 44, 45, 46]. The four representative color states (i.e., green, yellow, transparent, and blue; labeled as 1 to 4) from Figure 2c are displayed in Figure 2d. The PB‐Zn‐NiHCF devices induce reversible, independent color switching in four states because the two electrochromic electrode layers can be operated individually. Besides, this independent control mechanism allows the device to reproduce hue within its defined gamut, affirming its high color tenability. Notably, simultaneous coloring or bleaching of the dual electrochromic layers enables rapid transitions between states 2 and 4. In contrast, transitions between states 1 and 3 can be achieved through sequential activation of the two layers. Real‐time spectral analysis was conducted to evaluate the switching kinetics across various color states. For the blue‐transparent transition at 632 nm (1.8–0.6 V; Figure 2e), the devices exhibit a *t*_c_ of 9.2 s and a *t*_b_ of 15.4 s. Similarly, the yellow‐transparent transition at 420 nm (2.6–0.6 V; Figure 2f) reveals corresponding switching times of 8.0 s (*t*_c_) and 25.6 s (*t*_b_). In the NiHCF‐Zn‐PB ZECD, the electrochromic performances of the NiHCF and PB layers (e.g., cyclic voltammetry, coloration speed, coloration efficiency, optical contrast, and cycling stability) match those of their respective single‐layer devices (Figures S8, S9, S13, S16, and 1, 2; and Table S1). This indicates that integrating the two electrochromic layers does not introduce significant mutual interference; they operate independently, with no observable ionic competition or electrochemical coupling effects.

To elucidate the operational mechanism of this electrochromic platform, we analyzed the energy level transitions of Zn, PB, and NiHCF (Figure 2g). The pronounced potential gradients, approximately 1.12 V between Zn/PB and 1.45 V between Zn/NiHCF, serve as the driving force for spontaneous color switching. These gradients enable both self‐powered optical modulation and efficient redox‐modulated color switching. This Zn‐anode‐based electrochromic platform, featuring dual electrochromic layers, uniquely integrates intrinsic energy conversion with precise electrochemical control. Through internal potential differences, it directly transforms chemical energy into electrical output, autonomously triggering the bleaching process while simultaneously powering external minor appliances (Figure S17). This dual functionality showcases an excellent integration of electrochromic modulation with self‐powered micropower generation, operating entirely without external voltage input. Furthermore, electrochemical kinetics analysis confirms that the device operates through a hybrid mechanism combining diffusion‑controlled (battery‑type) and surface‑controlled (capacitor‑type) processes, as evidenced by *b*‐values exceeding 0.5 (Figure S18). The galvanostatic charge‐discharge (GCD) profiles at selected cycles (Figure S19) exhibit stable voltage plateaus with minimal polarization and consistent capacity retention, confirming the excellent electrochemical stability and reversibility of the Zn‐PB platform during prolonged cycling. The PB and NiHCF electrodes within the ZECD device exhibit a modest built‐in potential of 0.33 V, facilitating spontaneous electron flow from PB to NiHCF. When fully charged, these electrodes generate a built‐in voltage of 0.215 V (Figure 2h), arising from their internal potential difference. Integrated within a rocking‐chair electrochromic configuration, this built‐in voltage can supply sufficient power to illuminate an LED via a “Joule thief circuit” (Figure 2i), effectively functioning as a self‐sustained, battery‐like electrochromic system. To further demonstrate the generality of this strategy, we constructed spontaneous color‐switching systems based on WO_3_, including PB‐Zn‐WO_3_ (Figure S20) and NiHCF‐Zn‐WO_3_ (Figure S21) configurations, both of which exhibited self‐powered color switching driven by the intrinsic redox potential differences, confirming the broader electrochromic applicability of our approach.

### 2.4 Multilevel Information Encryption and Decryption of PB‐Zn‐NiHCF ZECDs

**FIGURE 3:**
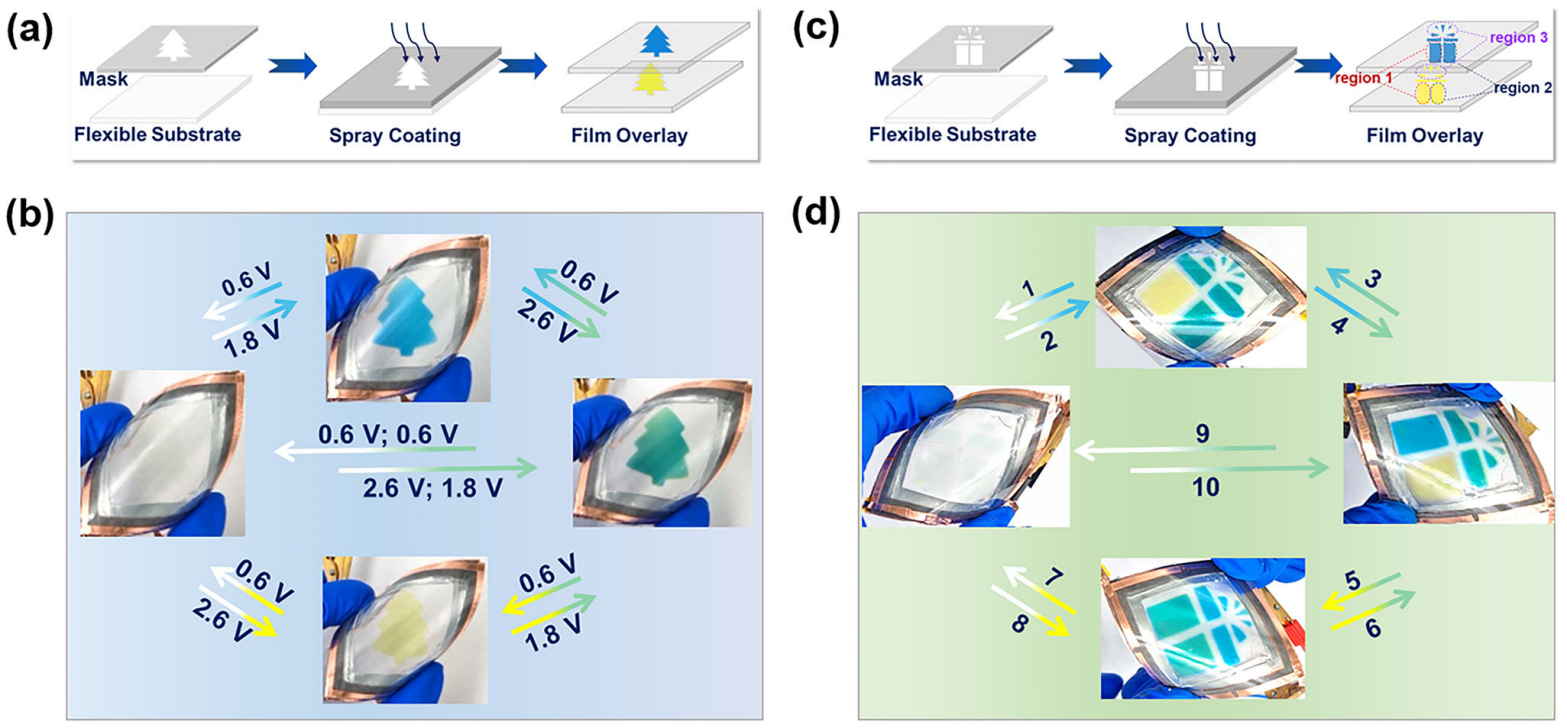
Demonstration of flexible patterned PB‐Zn‐NiHCF ZECDs from transparent to multicolor states. (a) Schematic of the fabrication process and (b) digital photographs of the patterned multicolor electrochromic system. (c) Schematic of the fabrication process and (d) digital photographs of the multi‐region tunable patterned multicolor electrochromic system.

As a proof of concept, we designed patterned multicolor electrochromic systems to showcase the 2D CIE color tunability of our PB‐Zn‐NiHCF ZECDs, highlighting their promise for practical display applications. We fabricated “tree‐like” electrochromic films by spray‐coating the aqueous suspensions of PB and NiHCF nanoparticles onto two flexible ITO/polyethylene terephthalate (PET) substrates through a patterned mask and then stacking them together (Figure 3a). The flexible PB‐Zn‐NiHCF ZECD device exhibited excellent electrochromic performance (Figure S22) and mechanical stability under deformation (Figure S23). This device exhibits excellent mechanical flexibility, maintaining stable electrochromic performance under deformation. The system demonstrates reversible color transitions across transparent, blue, yellow, and green states through independent control of each film (Figure 3b), validating its dynamic color control in patterned configurations. To further showcase the versatility of our platform, we made a “gift box”‐shaped electrochromic device (Figure 3c). This design employs the same spray‐coating method on two films as the “tree‐like” device, but with three independently addressable electrode regions. This architecture facilitates precise and localized color modulation across distinct sections of the device, enabling reversible transitions from transparent to various color states (i.e., yellow, blue, and green; Figure 3d). Video S2 demonstrated the rapid color‐switching process in selected regions of the “gift box” between green and yellow. These transitions can be triggered independently or simultaneously across the selected areas (Figure S24). The resulting prototype demonstrates broad tunability across the 2D CIE color space with localized control, enabling dynamic switching between chromatic states. This capability holds promise for both high‐performance displays (e.g., dynamic signage or wearable electronics) and advanced optical encryption, where color patterns serve as reconfigurable security features for anti‐counterfeiting or covert data storage.

As information security becomes increasingly critical, advanced encryption technologies are essential for safeguarding assets and ensuring data integrity. The PB‐Zn‐NiHCF display system demonstrates exceptional reversible color‐switching capabilities, making it highly promising for applications in secure data encryption. Its broad color gamut and operational versatility render it especially effective for covert optical data encoding. As a proof of concept, we showcase its reusable multi‐mode encryption and decryption potential, leveraging its tunable chromatic properties to achieve multi‐level information display and secure communication.

**FIGURE 4:**
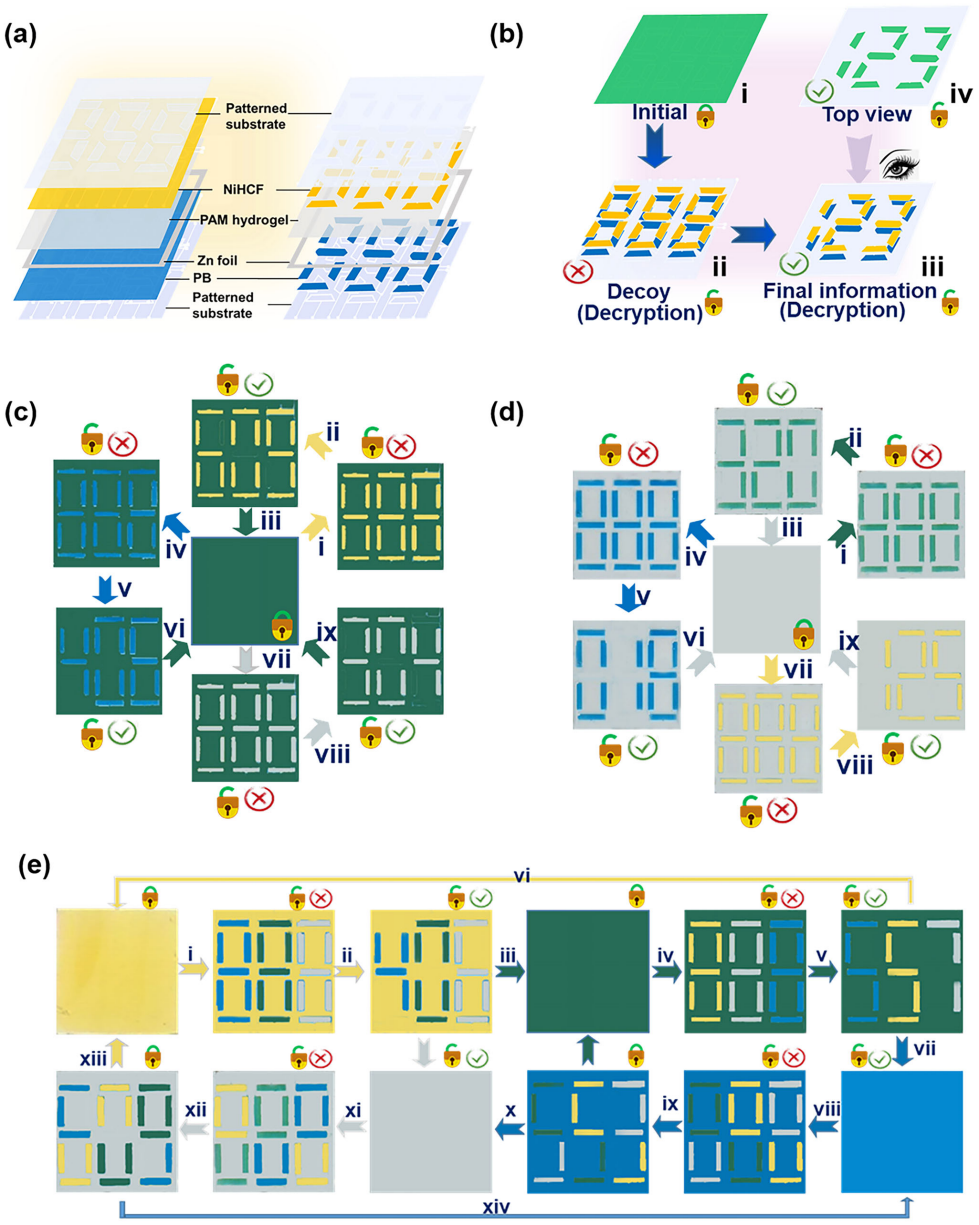
Multilevel information encryption and decryption applications. (a) Illustration of the PB‐Zn‐NiHCF electrochromic display structure. The display was composed of a Zn foil sandwiched between the two electrodes, with each side along with a precisely engineered substrate. (b) The electrochromic display enabled advanced information encryption by using a decoy mechanism that discloses the final information. (c‐e) Real photographs of the PB‐Zn‐NiHCF devices. A multi‐level information display process utilized (c) colored/(d) bleached NiHCF‐PB layers on a (c) green/(d) transparent background, enabling selective visualization of numerals in blue, yellow, and (c) transparent/(d) green states. (e) A Multi‐level, multi‐color information display process showed different color schemes in a single PB‐Zn‐NiHCF device.

Figure S25 shows the details of the seven segments of a number eight, which were laser‐etched onto an ITO/glass substrate. There are therefore seven patterned regions for the seven segments, and the rest area is a continuously connected, a non‐patterened region. This design enables independent control of each segment and the background, and thus allows individual color manipulation while preserving other functionalities. As illustrated in Figure 4a, finely patterned three digits of 888 were fabricated with every segment independently controlled/addressed. Subsequently, NiHCF and PB materials were uniformly sprayed across both patterned and non‐patterned regions, with NiHCF applied as the top layer and PB as the bottom layer of the device. This multi‐layer structure effectively embeds the initially invisible yellow/blue 888 within the device. The electrochromic display subsequently presents a uniform green hue due to the color overlay effect. Our precisely engineered electrochromic displays achieve high‐level information encryption by employing a decoy mechanism that gradually reveals the final information (Figure 4b).

In the first encryption level (Figure 4c,i–iii), the device displays a uniform green background resulting from the optical overlay effect between the top NiHCF (yellow) and bottom PB (blue) layers. The NiHCF electrode remains electrochemically stable under unbiased conditions. Applying 0.6 V to the patterned region of the bottom PB electrode layer induces electrochemical reduction, turning PB from blue to colorless and revealing the underlying yellow 888—unlocking the first level of encrypted information. Video S3 shows that the green device displays 888 in green against a yellow background when voltage was applied to the precise sections. It should be noted, however, that this 888 acts as a decoy and is distinct from the actual information. Subsequently, applying 1.8 V to the targeted segments of patterned regions in the bottom PB electrode layer induces electrochemical coloring in the transparent regions, rendering them blue (Figure S26). Through this sequential voltage application strategy and color overlay effect, the second layer of hidden information (the true 609 in yellow color against a green background) is revealed, demonstrating the system's capacity for multistage optical encryption. To re‐encrypt the device, 2.6 V/1.8 V were applied to the bleached segments of NiHCF/PB layers, respectively, and their original yellow/blue states were effectively restored to display a uniform green device. This process effectively re‐encrypts the device, erasing the revealed information and restoring the system to its original state, thus completing a fully reversible encryption and decryption cycle (Figure 4c,i–iii). Similarly, when the PB electrode remains unbiased, applying 0.6 V to patterned regions of the top NiHCF layer bleaches the yellow layer, revealing a blue 888 against a green background. Further selective coloration of the targeted segments of patterned regions in the top NiHCF layer unveils the hidden number 406 in blue color against a green background, offering an alternative decryption pathway (Figure 4c, iv–vi).

When all the patterned and non‐patterned regions of both NiHCF and PB electrodes are simultaneously bleached by applying 0.6 V, the device is in a colorless state, and all information is encrypted (the central photograph in Figure 4d). Applying oxidation voltages to patterned regions of the two NiHCF (2.6 V) and PB (1.8 V) electrodes, the device displays a decoy pattern consisting of a green 888 numeral against a transparent background (Figure S27). This visual effect originates from the maintained spectral overlap of NiHCF and PB within the patterned regions (Figure 4d, i). Further selective and simultaneous bleaching of PB and NiHCF reveals a green 320 against a colorless background, thereby disclosing the intended encrypted information. (Figure 4d, ii). Complete bleaching of all regions on both electrodes resets the device to a fully transparent state, thereby re‐concealing all previously revealed information (Figure 4d, iii). Alternatively, applying 1.8 V exclusively to the patterned regions of the PB layer restores a blue 888 on a colorless background (Figure 4d, iv), while applying 2.6 V to the corresponding NiHCF 888 regions yields a yellow 888 (Figure 4d, vii). Subsequent selective bleaching reveals the target information, a blue 018 (with PB active and NiHCF bleached; Figure 4d, v) or a yellow 125 (with NiHCF active and PB bleached; Figure 4d, viii) with a transparent background. These results highlight the system's versatile information retrieval capability through precise electrochemical control. Selective bleaching and coloring of different electrode units enables dynamic multicolor numeral displays against various backgrounds. For example, the system can present green (553), blue (406), or colorless (666) numerals on a yellow background (Figure S28a). Similarly, it can display green (229), yellow (557), or colorless (315) numerals against a blue background (Figure S28b). This versatility manifests the platform's advanced optical programmability and spatially controlled electrochemical modulation.

To further demonstrate its encryption capabilities, we also designed a dynamic electrochromic decryption system that performs stepwise decryption via multistep color switching. The device is capable of simultaneously displaying 888 in three distinct colors (blue, green, and transparent, respectively with a yellow background; Figures 4e,i, and S29), which can be selectively decrypted into 406 while retaining the same color scheme (Figure 4e,ii). For the second encryption level, the decrypted color scheme exhibits increased complexity. The two‐step decryption strategy guarantees that encoded information remains securely concealed until the correct electrochemical activation sequence is applied. Further oxidative bias enables the device to be reversibly switched to a uniform green state, effectively re‐encrypting the display and concealing the previously revealed data (Figure 4e,iii). To enhance encryption depth, we introduced a device featuring a green background and a multicolor 888 (yellow, transparent, and blue, respectively; Figure 4e,iv). The 888 can unexpectedly transform into a color‐programmed 557 (blue, yellow, and transparent, respectively; Figure 4e,v), achieving a higher‐level decoding through the rearrangement of color sequences (Figure S30). This reconfiguration represents the third encryption level. The independent operability of ZECDs, combined with the ingeniously engineered device architecture, enables a more refined encryption mechanism. Notably, the 888 composed of dual‐color segments within one digit (green‐transparent, yellow‐green, transparent‐yellow) can be selectively decrypted into 553 through precise electrochemical control while preserving the original color arrangement within each corresponding digit (Figure 4e,viii–ix), demonstrating an advanced fourth encryption level achieved through precise electrochemical control. Inspiringly, the system allows dynamic color reconfiguration within individual digits, enabling selective decryption into letters with modified internal color arrangements. For instance, the 888 (yellow‐green, green‐blue, blue‐yellow) can be transformed into 609 with rearranged segment colors (blue‐yellow, yellow‐green, green‐blue; Figure 4e,xi–xii), representing the most advanced fifth encryption level in this work (Figure S31). Besides, the various encryption modes in Figure 4e can be converted arbitrarily. This finding lays the groundwork for the rational design and scalable fabrication of intelligent, reconfigurable encryption devices.

Our PB‐Zn‐NiHCF ZECD information encryption system offers distinct advantages over recent state‐of‐the‐art electrochromic encryption platforms, particularly in terms of encryption efficiency, security level, and dynamic tunability (Table S2). Our system introduces a multilayered encryption architecture with a dynamic multi‐stage visual transition process (blank, decoy pattern, and final information), ensuring that data is securely protected until a full decryption is authorized. This mechanism ensures staged access and controllable decryption that significantly enhances information security by maintaining content in a “decoy display” state before an authorized disclosures. Compared to conventional static methods, this approach achieves superior security through multi‐stage authentication, real‐time color modulation, and adjustable encryption levels tailored to specific needs. The system strengthens these features with precisely tunable, responsive color‐switching kinetics, enabling dynamic, on‐demand encryption without predefined design constraints and allowing active control during operation. The layered pattern realizes high‐purity, multilevel visual displays, reversible electrochromic write/erase cycles, and improved encryption performance, advancing both visual appearance and data protection.

**FIGURE 5:**
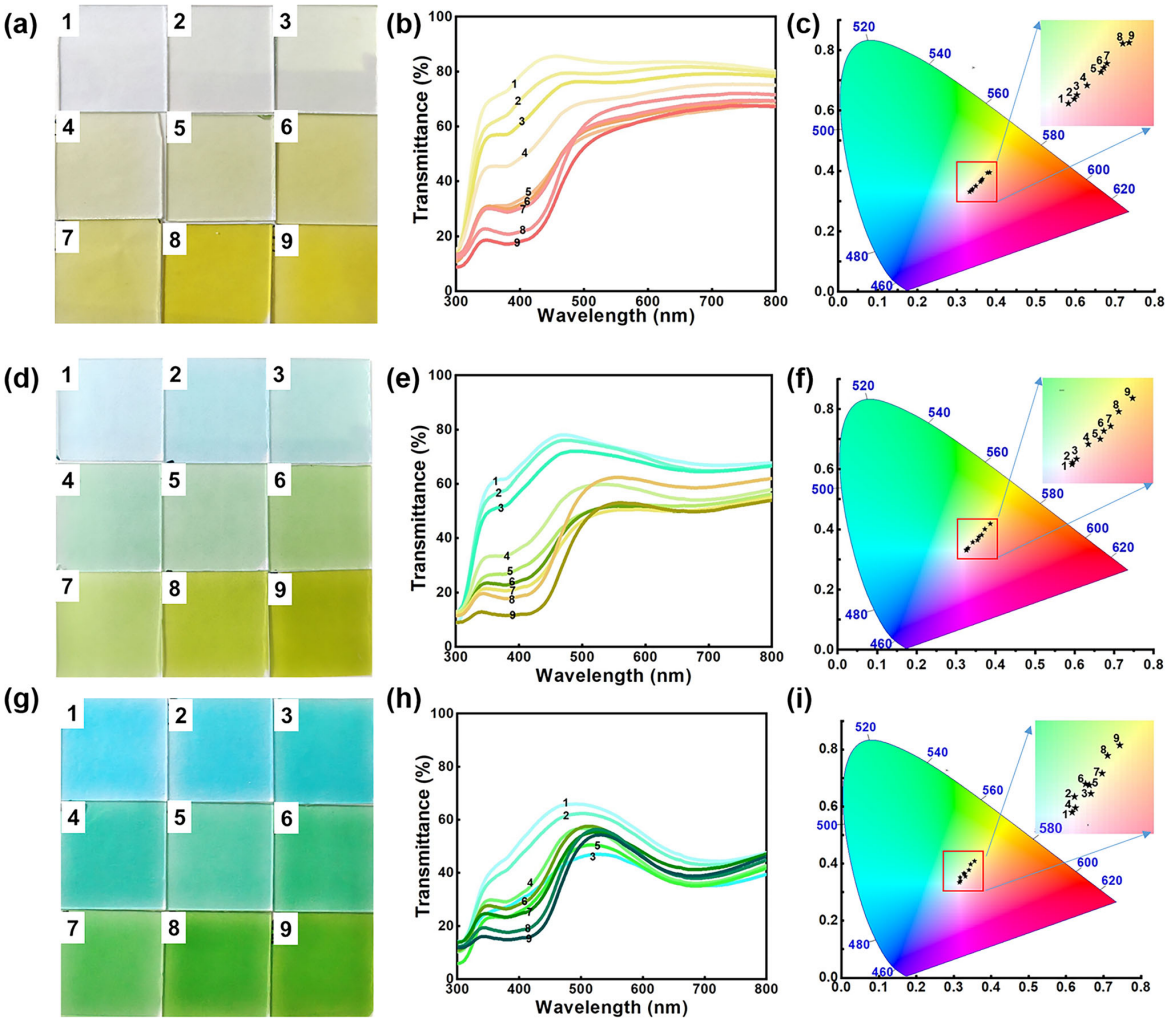
More color variations of electrochromic films with varying PB/NiHCF ratios for information security applications. Optical properties of the films with PB: NiHCF ratios from 1:10 to 1:400: (a) photographs, (b) optical transmittance spectra, and (c) CIE coordinates (numbers correspond to colors in a) across light‐to‐dark yellow states. Optical properties of the films with PB: NiHCF ratios from 5:10 to 5:400: (d) photographs, (e) transmittance spectra, and (f) CIE coordinates for light blue/blue‐green/yellow states. Optical properties of the films with PB: NiHCF ratios from 10:10–10:400: (g) photographs, (h) transmission spectra, and (i) CIE coordinates across blue/blue‐green/green states. All ratios demonstrate nine distinct color states, with the labeling numbers corresponding to colors in each panel.

While color overlay strategies have been employed in ECDs to achieve multicolor displays, their development is limited by challenges such as imprecise color control, low spectral purity, and restricted color gamut. To address these challenges, we further developed a nanoparticle‐blending strategy using PB and NiHCF, allowing precise color modulation through PB/NiHCF mixing ratio while maintaining high color purity and generating a broad spectrum of vivid hues (Figure S32). Electrochromic films were fabricated via spray‐coating the mixed PB and NiHCF nanoparticles onto ITO/glass substrates. As shown in Figure 5, this system demonstrates 27 distinct representative color states, highlighting its potential for advanced multicolor ECD applications. The transmittance of the films at 420 nm gradually declines as the PB/NiHCF ratio increases from 1:10 to 1:400 (samples 1–9), accompanied by a color transition from light yellow to yellow (Figure 5a–b). Corresponding CIE 1931 color coordinates show a broad distribution within the yellow region (Figure 5c). Further increasing the mixing ratio from 5:10 to 5:400 continues to reduce transmittance at 420 nm, producing a progressive transition from light blue to blue‐green and ultimately to yellow (Figure 5d–e), with CIE color coordinates confirming extensive coverage across the blue, green, and yellow areas (Figure 5f). At higher ratios (10:10 to 10:400), transmittance at both 420 and 632 nm decreases, resulting in transitions through blue, blue‐green, and green hues (Figure 5g–h), again corroborated by the corresponding CIE coordinates (Figure 5i). Adjusting the PB/NiHCF ratio enables the device to offer an expanded palette of color options, with each hue functioning as an independent, reversibly switchable electrochromic unit. This capability facilitates the creation of multi‐channel systems for information display and encryption, significantly enhancing both the complexity and security of encoded data. Moreover, the independent control of each color state allows the device for self‐powered chromic switching, eliminating the need for an external power source. This feature further broadens its applications across anti‐counterfeiting technologies, wearable electronics, and intelligent display systems.

## 3 Conclusion

In summary, we developed PB‐Zn‐NiHCF ZECDs for information encryption applications. The PB‐Zn‐NiHCF ZECDs of 5 × 5 cm^2^ demonstrate outstanding dynamic visible modulation (Δ*T* = 53.6% at 632 nm and Δ*T* = 31.5% at 420 nm) and rapid switching times (*t*_c_ = 15.4 s and *t*_b_ = 9.2 s between green and transparent at 632 nm). These ZECDs represent a significant advancement in 2D CIE color space modulation, offering four distinct visual states—transparent, blue, green, and yellow. Through a meticulously engineered multi‐area addressable conductive substrate configuration, the ZECDs enable precise, localized, and dynamic control of electrochromic displays. This innovation opens new possibilities for adaptive encryption and identity verification. Unlike traditional static and one time‐configured methods, our approach delivers enhanced security via multi‐stage authentications, real‐time color modulation, and customizable encryption levels tailored to specific needs.

However, we acknowledge certain limitations that point toward future research directions. First, the current prototype relies on an external power supply and driving electronics for electrochromic switching and logic control, which constrains its portability and autonomy in potential stand‐alone applications. Future integration of low‐power, embedded control circuits would enhance compactness but underscores the need for sustainable on‐board power solutions, such as integrated energy harvesters (e.g., triboelectric nanogenerators). Second, the long‐term cycling stability under extreme environmental conditions (e.g., high humidity, temperature fluctuations) remains to be thoroughly evaluated to ensure reliability for commercial deployment. Future work will be on exploring novel nanostructured or composite electrochromic materials to broaden the color gamut and improve switching durability, alongside developing flexible, stretchable, or even biodegradable substrates to expand applicability in wearable and sustainable electronics. Another effort is to investigate the integration of energy harvesting modules, such as nanogenerators, thin‐film solar cells, or bioenergy batteries, to create self‐powered authentication systems that operate independently of external power sources. These would significantly enhance the deployability, sustainability, and user experience of next‐generation secure displays. In high‐end consumer electronics and anti‐counterfeiting traceability, this system can be applied to authentication labels for luxury goods, pharmaceuticals, and premium liquors. During verification via designated devices, the label undergoes a dynamic transition from a blank state to a three or more‐digit decoy code, and finally reveals a unique authentication identifier. This multi‐stage decryption process is extremely difficult to replicate, establishing a direct and tamper‐proof physical gateway for brand protection and consumer trust. The technical foundation of this platform, especially as it evolves toward greater autonomy and robustness, can address the increasing market demand for dynamic, item‐level anti‐counterfeiting solutions. We envision a new generation of flexible ZECDs that not only improve display performance but also strengthen information security. Our goal is to drive electrochromic technology toward smarter, more secure, and highly adaptable applications.

## Author Contributions

**Feifei Zhao**: conceptualization, methodology, data curation, software, formal analysis, visualization, investigation, and writing – original draft. **Bingkun Huang**: methodology, data curation, and investigation. **Kaili Gong**: investigation, data curation. **Bin Wang**: methodology, investigation. **Anni Sun**: investigation, data curation. **Yilin Liu**: data curation, investigation. **Wu Zhang**: validation, visualization, and writing – review and editing. **Jingwei Chen**: resources, visualization, and validation. **Haizeng Li**: conceptualization, funding acquisition, methodology, project administration, supervision, and resources. **William W. Yu**: supervision, resources, project administration, writing – review and editing, investigation, methodology, validation, visualization, and funding acquisition.

## Conflicts of Interest

The authors declare no conflicts of interest.

## Supporting information




**Supporting File 1**: anie72192‐sup‐0001‐VideoS1‐S3.zip.

**Supporting File 2**: anie72192‐sup‐0004‐SuppMat.docx.

## Data Availability

The data that support the findings of this study are available from the corresponding author upon reasonable request.
