# Supplementary material for: Multi‐Color Flexible Electrochromic Device for Smart Anti‐Counterfeiting
Source: Angew Chem Int Ed Engl. 2026 Apr 13;65(22):e6863859. doi: 10.1002/anie.6863859 (PMC13206437; doi:10.1002/anie.6863859)
Supplement: Supplementary file 2 — Supporting File 2: anie72192‐sup‐0004‐SuppMat.docx. [file ANIE-65-e6863859-s001.docx]

**Multi-Color Flexible Electrochromic Device for Smart Anti-Counterfeiting**

Feifei Zhao, Bingkun Huang, Kaili Gong, Bin Wang, Anni Sun, Yilin Liu, Wu Zhang*, Jingwei Chen*, Haizeng Li*, William W. Yu*

F. Zhao, K. Gong, Anni Sun, Yilin Liu, W. W. Yu
School of Chemistry and Chemical Engineering, Ministry of Education Key Laboratory of Special Functional Aggregated Materials, Shandong Key Laboratory of Advanced Organosilicon Materials and Technologies, Shandong University, Jinan 250100; Shandong Provincial Key Laboratory for Science of Material Creation and Energy Conversion, Science Center for Material Creation and Energy Conversion, Shandong University, Qingdao 266237, China
Email: wyu6000@gmail.com

B. Wang, B. Huang, H. Li
Institute of Frontier and Interdisciplinary Science, Shandong University, Qingdao 266237, China
Email: haizeng@sdu.edu.cn

H. Li

Deceased.

W. Zhang

Ultrafast Optics and Nanophotonics Laboratory, Department of Electrical and Computer Engineering, University of Alberta, Edmonton, AB, T6G 2V4, Canada

Email: wzhang1@ualberta.ca

J. Chen

School of Materials Science and Engineering, Ocean University of China, Qingdao, China

Email: chenjingwei@ouc.edu.cn

**Experimental section**

**Materials:** All solvents and chemicals were of analytical grade and were used without further purification. Zinc foil (Zn, 99.9%), potassium chloride (KCl, 99.8%), potassium hexacyanoferrate (III) (K_3_[Fe(CN)_6_], 99.5%), iron(II) chloride tetrahydrate (FeCl_2_·4H_2_O, 99%), nickel(II) chloride hexahydrate (NiCl_2_·6H_2_O, 98%), ammonium persulfate ((NH_4_)_2_S_2_O_8_, 99.9%), zinc perchlorate heptahydrate (Zn(ClO_4_)_2_·7H_2_O) and tetramethyl-ethylenediamine (TEMED) were all purchased from Macklin Biochemical Technology Co. Ltd. Acrylamide (AM) and N, N-methylenebisacrylamide (MBAA) were purchased from Sigma-Aldrich. Ultrapure water (>18 MΩ cm) from a YETOP system (YTUP-30) was utilized throughout the experiments. Indium tin oxide-coated glass (ITO/glass, transmittance >77%, sheet resistance ~7 Ω/sq^-1^) was purchased from Zhuhai Kaivo Glass Co. Ltd. Flexible indium tin oxide/polyethylene terephthalate (ITO/PET) films were provided by Qianyu Electronic Materials (Shenzhen) Ltd.

**Synthesis of NiHCF and PB nanoparticles:** In a typical synthesis of NiHCF nanoparticles, NiCl_2_·6H_2_O (2 mmol) and K_3_[Fe(CN)_6_] (2 mmol) were individually dissolved in 50 mL of distilled water. The K_3_[Fe(CN)_6_] solution was then added dropwise to the NiCl₂ solution under magnetic stirring. After 3 hours, a yellow precipitate formed and was collected via three rounds of centrifugation at 10,000 rpm for 10 minutes with acetone addition. The obtained precipitate was redispersed in appropriate deionized water. PB nanoparticles were prepared using the same procedure, substituting NiCl_2_·6H_2_O with FeCl_2_·4H_2_O.

**Fabrication of NiHCF and PB electrodes:** The ITO/glass was treated with a plasma cleaner for 60 seconds before spraying. Solutions of PB nanoparticles (0.2 mL, 32 mg/mL in ethanol) and NiHCF nanoparticles (0.5 mL, 10 mg/mL in ethanol) were then sprayed onto the cleaned ITO/glass (with an effective area of 3 x 3 cm^2^) at 55°C, respectively. In typical, the electrochromic electrodes were fabricated with additions of 100 μL PEDOT: PSS (1.4% in water), 5 μL, and 10 μL Mxene (0.035 mg/mL), respectively. Afterward, the PB and NiHCF electrodes were obtained by annealing at 65°C for 24 hours to improve the electrode properties.

**Fabrication of WO_3_ electrode:** The ITO/glass was treated with a plasma cleaner for 60 s before spraying. The WO_3_ cathode was fabricated via a pulsed electrodeposition technique. Electrodeposition was carried out in a three-electrode setup, with the ITO-coated substrate serving as the working electrode, an Ag/AgCl electrode as the reference, and a stainless steel mesh as the counter electrode. The precursor solution was prepared by dissolving 0.412 g of Na_2_WO_4_·2H_2_O in 100 mL of deionized water, followed by the addition of 0.8 mL of HClO_4_ and 0.26 mL of H_2_O_2_ under continuous magnetic stirring. WO_3_ films were deposited by applying a pulsed potential of -0.7 V for 0.2 s, followed by 0.1 V for 0.8 s, and repeating this cycle 2000 cycles.

**Preparation of PAM ionic** **hydrogel:** AM monomer (2.5 g) was dissolved in a 1.0 M KCl-Zn(ClO_4_)_2_ aqueous solution (0.9 M KCl-0.1 M Zn(ClO_4_)_2_, 7.5 mL) under stirring. The cross-linker MBAA (20 mg) and the initiator (NH_4_)_2_S_2_O_4_ (20 mg) were then added, followed by sonication and agitation until a transparent solution was obtained. Subsequently, 5 μL of TEMED was introduced to the solution. The well-mixed solution was immediately transferred to a glass mold and heated at 40 °C for 20 minutes, forming a 1 mm-thick ionic PAM hydrogel.

**Fabrication of ZECDs:** The Zn-NiHCF ZECDs were assembled using a NiHCF-coated ITO/glass as the electrochromic cathode, the PAM hydrogel attached to the NiHCF electrode as the electrolyte, and a zinc frame attached to blank glass as the anode. The Zn-PB ZECDs were fabricated similarly, substituting NiHCF-coated ITO/glass with PB-coated ITO/glass. The PB-Zn-NiHCF electrochromic displays were fabricated by sandwiching a thin zinc square frame between NiHCF-coated and PB-coated ITO/glass substrates, using PAM gel as the electrolyte. For the flexible version, the rigid ITO/glass was replaced with a specially designed ITO/PET flexible conductive substrate featuring independently addressable sections, while keeping all other parameters unchanged.

**Materials characterizations and electrochemical measurements:** The crystal structures and morphologies of the samples were examined using X-ray diffraction (XRD, Japan, Rigaku D/max-2200PC) and a field-emission scanning electron microscope (SEM, Quanta 250 FEG). All optical measurements were conducted using a UV-VIS-NIR spectrophotometer (Shimadzu UV-3600i Plus). All electrochemical measurements were performed using an electrochemical workstation (CHI-760E; CH Instruments, Shanghai, China) in a two-electrode configuration, with the electrochromic electrode serving as the working electrode, and a Zn foil as the counter electrode.


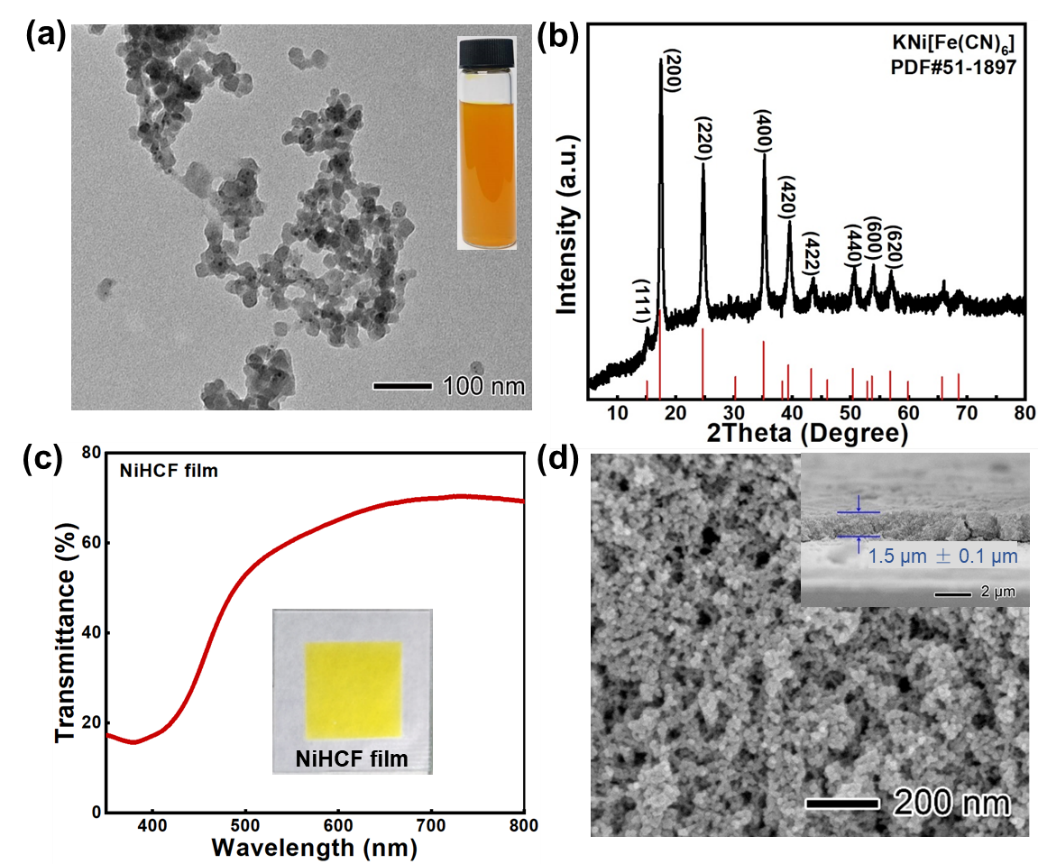


**Figure S1**. (a) TEM image (inset is a digital photograph of the NiHCF nanoparticle aqueous solution) and (b) XRD pattern of the NiHCF nanoparticles. (c) Optical transmittance spectrum, corresponding digital photograph, and d) surface and cross-sectional (inset) SEM images of the NiHCF film. We synthesized the NiHCF nanoparticles with an average size of 20 nm via a one-pot method (Figure S1a). XRD confirmed their cubic phase (Fm-3m, JCPDS No. 51-1897) (Figure 1b). We fabricated the NiHCF film by spray-coating aqueous NiHCF dispersions onto ITO/glass, yielding an electrode with 20.1% transmittance at 420 nm (Figure S1c). The rough and porous morphology (about 1.5 μm thick, Figure S1d) promotes ion transport, thereby boosting electrochromic performance (Figure S1d).


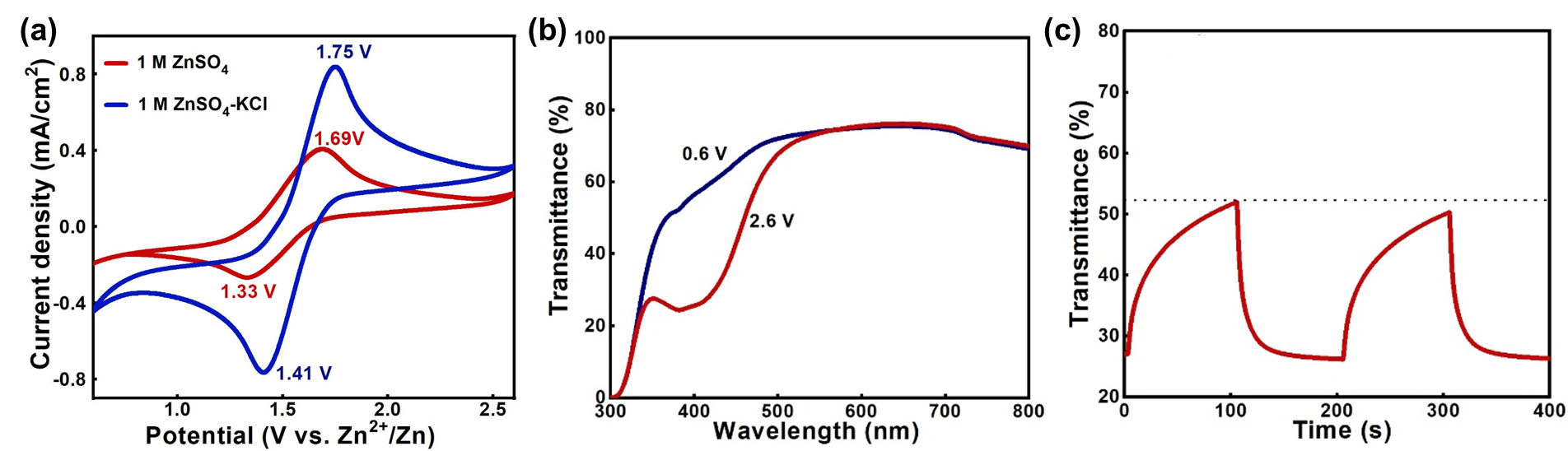


**Figure S2**. (a) Comparison of cyclic voltammograms of the NiHCF electrode in 1 M ZnSO_4_, and 1 M KCl-ZnSO_4_ electrolyte systemat a scan rate of 50 mV s^-1^ over a voltage range of 0.6-2.6 V. (b) Optical transmittance spectra and (c) real-time transmittance changes measured at 420 nm in 0.6-2.6 V of the Zn-NiHCF device with 1 M KCl-ZnSO_4_ aqueous electrolyte. The Zn-NiHCF device demonstrated limited cycling stability in aqueous Zn^2+^-K^+^ electrolyte, in agreement with the performance degradation observed in Figure S9b.


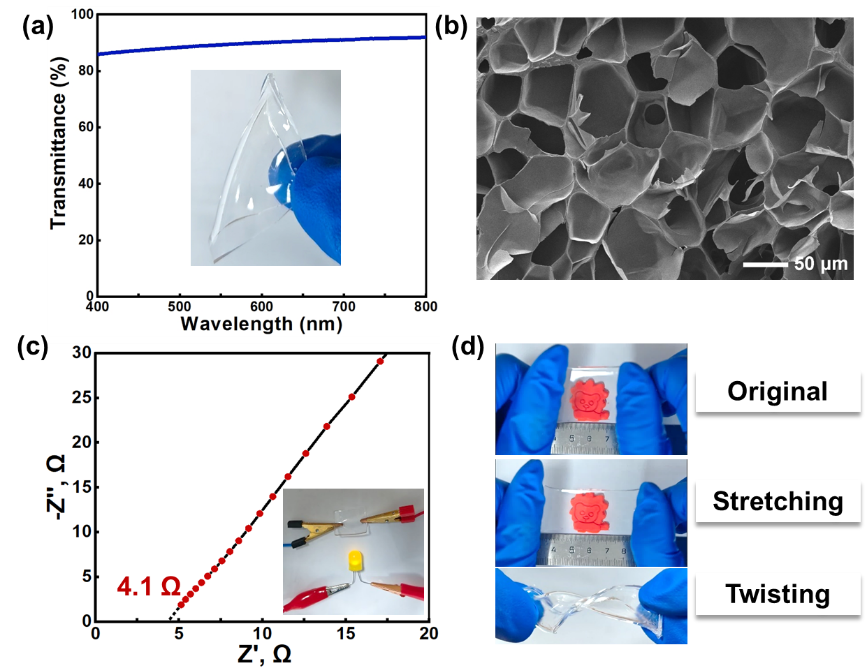


**Figure S3**. (a) Optical transmittance spectrum (inset: flexible PAM photograph), (b) SEM image, and (c) AC impedance spectrum of the PAM hydrogel (inset demonstrates LED illumination by using the PAM hydrogel as a conductor). (d) Digital photographs of the PAM hydrogel electrolyte showing its stretchability and twistability. The AC impedance spectrum of the PAM hydrogel film shown in Figure S3c yields an ionic conductivity (σ) of 2.4 S m^-1^, calculated using σ = L/(R × S),^[1-2]^ where L is the film thickness (cm), S is the electrode contact area (cm^2^), and R is the bulk resistance derived from the x-axis intercept.

$$\text{σ=}\frac{\text{L}}{\text{R × S}}\text{ = 0.1 cm/(4.1 Ω×1 cm}\text{2}\text{) = 0.024 cm}\text{-1 }\text{= 2.4 S m}\text{-1}$$

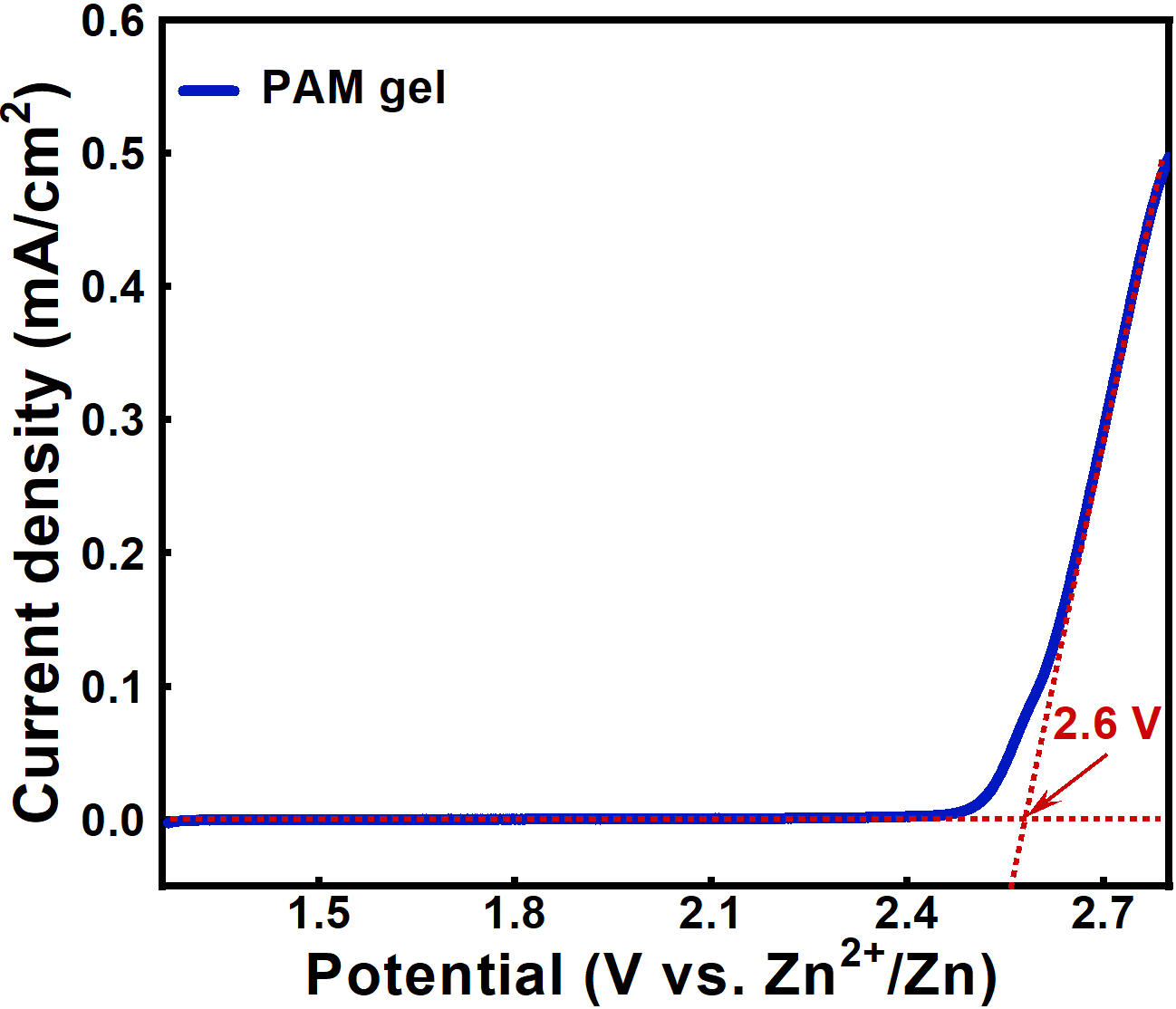


**Figure S4**. Linear sweep voltammogram (LSV) was obtained from the PAM gel electrolyte at a scan rate of 10 mV/s. The result indicates an electrochemical stability window of approximately 2.6 V for the electrolyte.


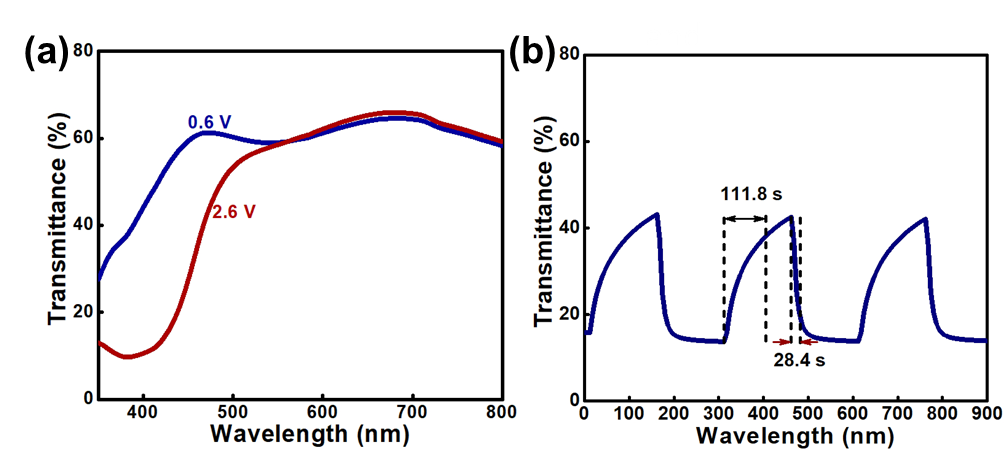


**Figure S5**. Electrochromic performance of the Zn-NiHCF device without conductive materials. (a) Optical transmittance spectra and (b) real-time transmittance changes measured at 420 nm in a 0.6−2.6 V window. The pristine NiHCF electrochromic materials-based Zn-NiHCF device showed slow response times, with a bleaching time (t_b_) of 111.8 s and a coloration time (t_c_) of 28.4 s.


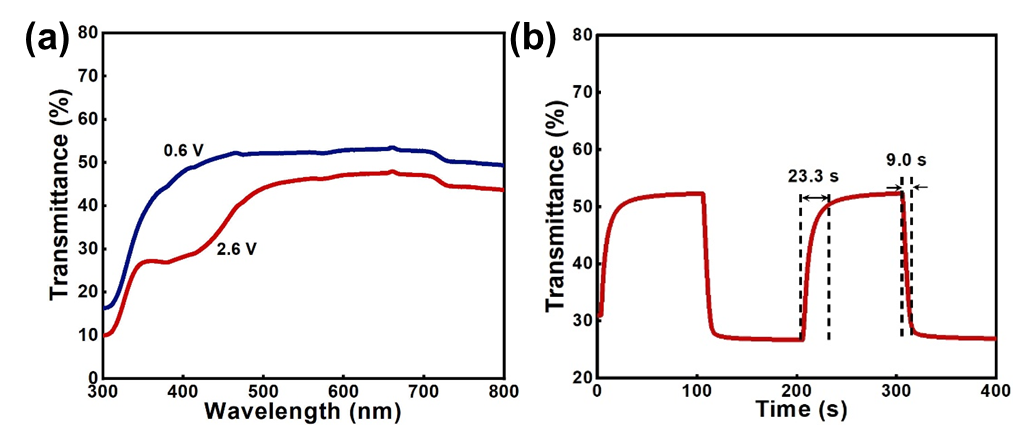


**Figure S6**. Electrochromic performance of the Zn-NiHCF device with conductive PEDOT:PSS. (a) Optical transmittance spectra and (b) real-time transmittance changes measured at 420 nm in a 0.6-2.6 V window. We added 50 μL PEDOT:PSS (i.e., Poly (3,4-ethylenedioxythiophene):poly (styrenesulfonate)) to the typical NiHCF electrochromic materials to enhance the conductive properties. The Zn-NiHCF device exhibited relatively inferior optical modulation (25.6% at 420 nm) and slow response times, with a bleaching time (t_b_) of 23.3 s and a coloration time (t_c_) of 9.0 s.


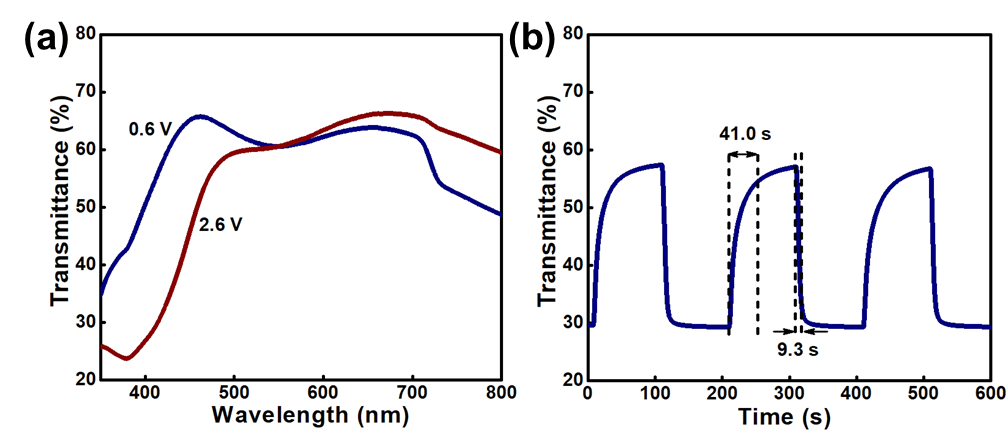


**Figure S7**. Electrochromic performance of the Zn-NiHCF device with 5 μL conductive Mxene. (a) Optical transmittance spectra and (b) real-time transmittance changes measured at 420 nm in a 0.6 V-2.6 V window. We added 5 μL Mxene to the typical NiHCF electrochromic materials to enhance the conductive properties. The Zn-NiHCF device showed relatively slow response times, with a bleaching time (t_b_) of 41.0 s and a coloration time (t_c_) of 9.3 s.


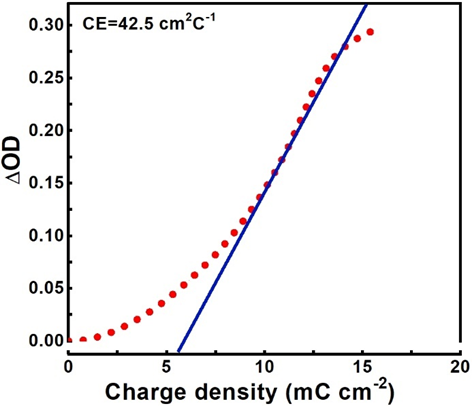


**Figure S8**. Coloration efficiency of the Zn-NiHCF at 420 nm. The calculated CE value of Zn-NiHCF is about 42.5 cm^2^ C^-1^.


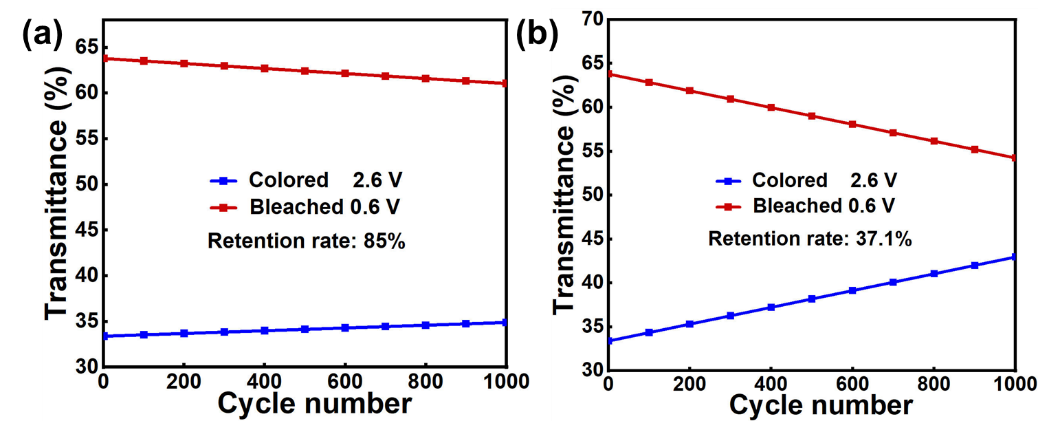


**Figure S9**. Cycling stability performance of the Zn-NiHCF device in a 0.6−2.6 V window color-switching using (a) 1 M Zn^2+^-K^+^ PAM hydrogel electrolyte and (b) 1 M Zn^2+^-K^+^ aqueous electrolyte. Impressively, the Zn-NiHCF device maintains 85% of its original optical contrast even after 1000 electrochemical cycles (Figure S9a), demonstrating exceptional long-term durability and stability. This performance is markedly superior to that of the Zn-NiHCF system employing an aqueous electrolyte, which exhibits a significantly lower retention of only 37.1% under identical cycling conditions (Figure S9b). The enhanced cycling stability of the Zn-NiHCF device can be attributed to the optimized gel electrolyte composition and improved interfacial compatibility, which collectively contribute to reduced degradation and sustained electrochromic functionality over prolonged operation.

**
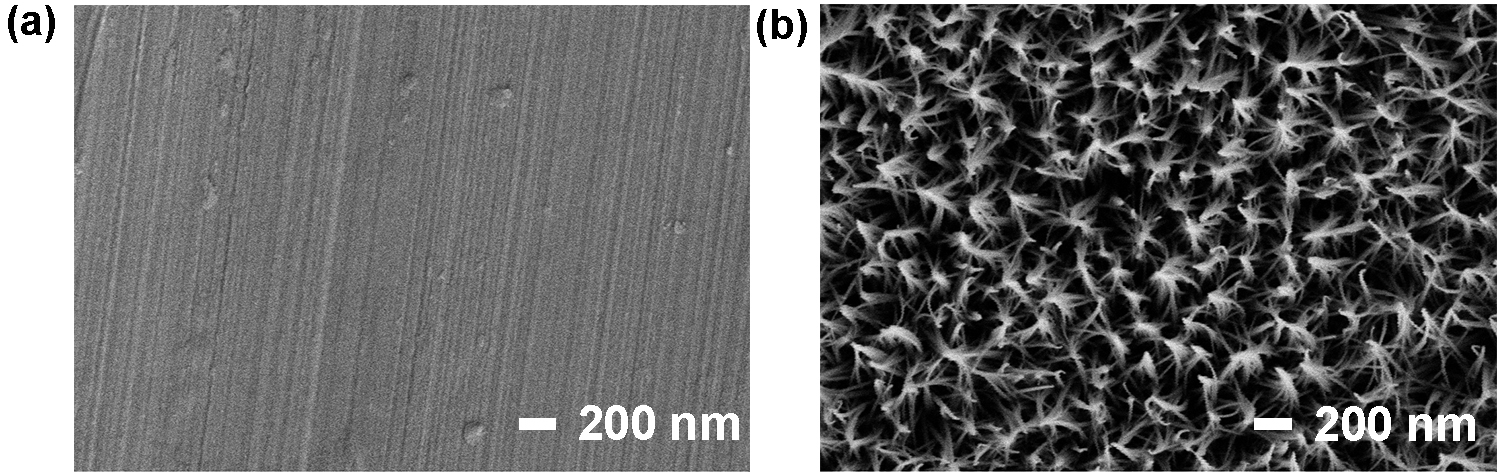
**

**Figure S10**. SEM images of (a) the pristine Zn foil and (b) the Zn foil retrieved after 1000 cycling cycles, showing the presence of Zn dendrites. The degradation in electrochemical performance of the cycled device may be attributed to the formation of zinc dendrites.

**
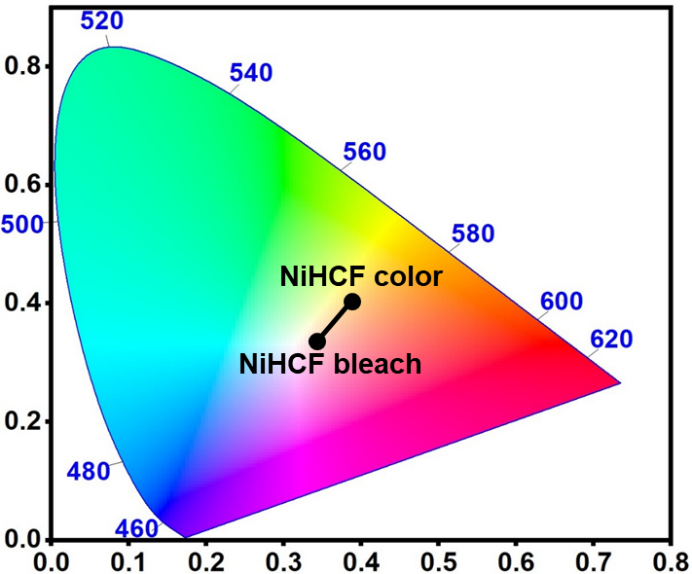
**

**Figure S11**. Single-layer Zn-NiHCF devices demonstrate one-dimensional linear color transitions. Conventional single-material-based devices (Zn-NiHCF devices) exhibited linear color transitions within a one-dimensional color space, restricting their achievable color gamut.


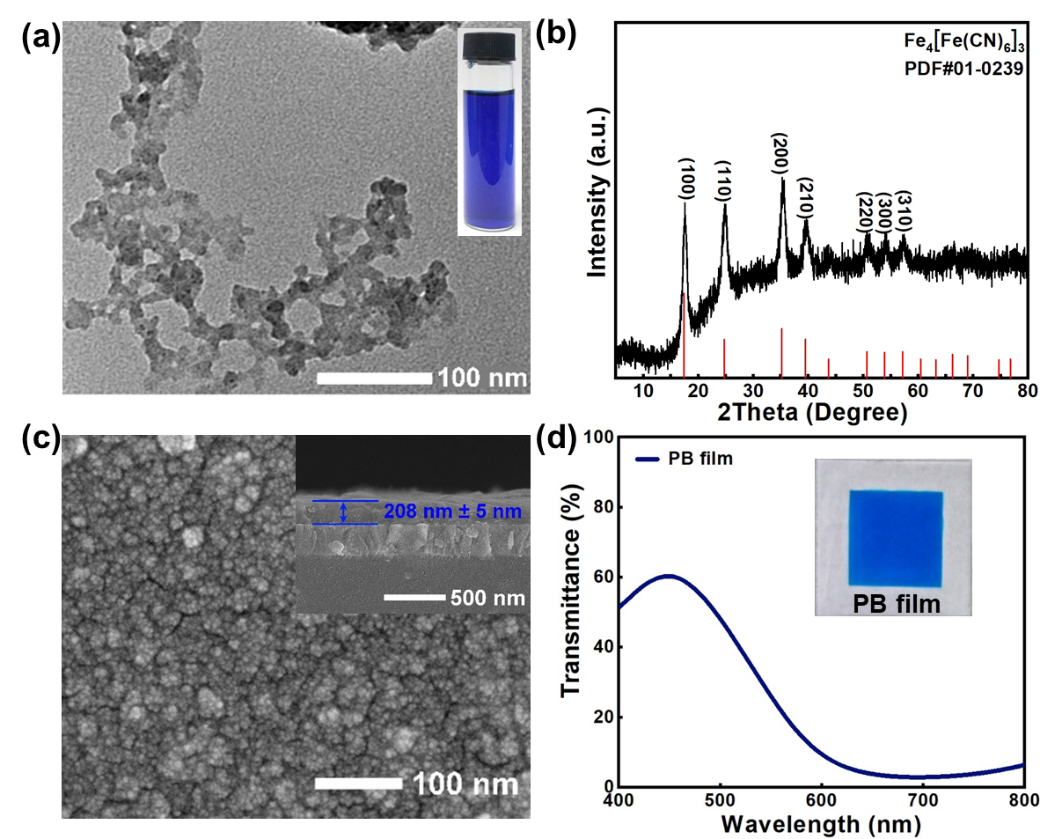


**Figure S12**. (a) TEM image (inset is a digital photograph of the PB nanoparticle aqueous solution) and (b) XRD pattern of the PB nanoparticles. (c) Surface and cross-sectional (inset) SEM images, and (d) optical transmittance spectrum of the corresponding digital photograph (inset) of the PB film. We synthesized the blue PB nanoparticles with an average size of 10 nm via a one-pot method (Figure S12a). XRD confirmed their cubic phase (Fe_4_[Fe(CN)_6_]_3_, PDF#01-0239) (Figure 1b). We fabricated the PB film with a thickness of approximately 208 nm by spray-coating PB aqueous solution onto ITO/glass, yielding an electrode with 5% transmittance at 632 nm (Figure S12c-d).


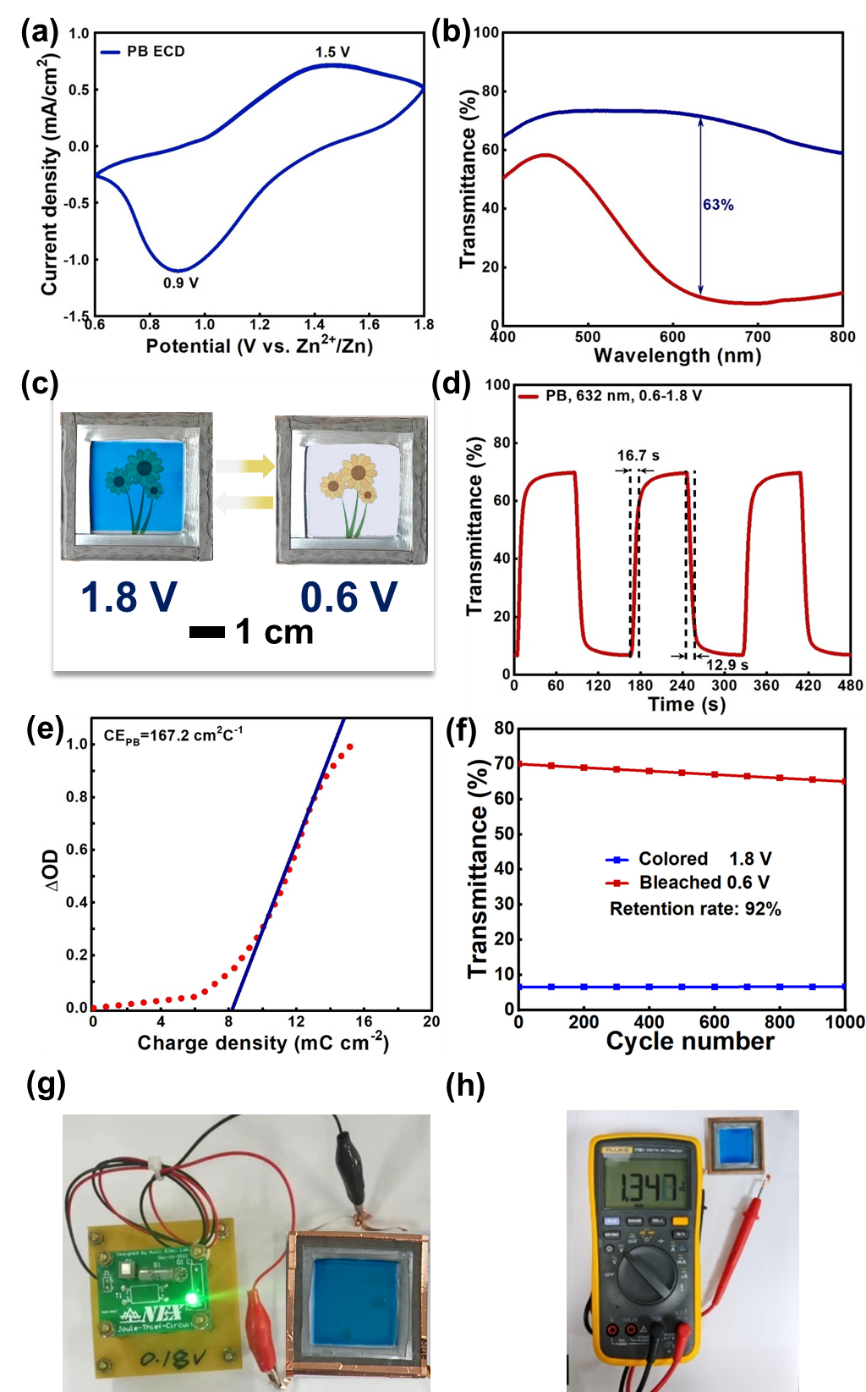


**Figure S13**. Electrochromic performance of the Zn-PB ZECD prototype device. (a) Cyclic voltammogram of the Zn-PB ZECD in a PAM hydrogel electrolyte system containing 1 M Zn(ClO_4_)_2_-KCl at a scan rate of 50 mV s^-1^ over a voltage range of 0.6−1.8 V. (b) Optical transmittance spectra and (c) corresponding digital photographs of the device (active area 3 × 3 cm^2^) in the colored and bleached states. (d) Real-time transmittance changes measured at 632 nm in the 0.6−1.8 V window. (e) Coloration efficiency of the PB electrode at 632 nm. (f) Zn-PB device retains 92% of its initial optical contrast after 1000 cycles. (g) An LED powered by the device in colored state via a “Joule thief circuit”. (h) Digital photograph depicting the device with an OCP of 1.347 V.


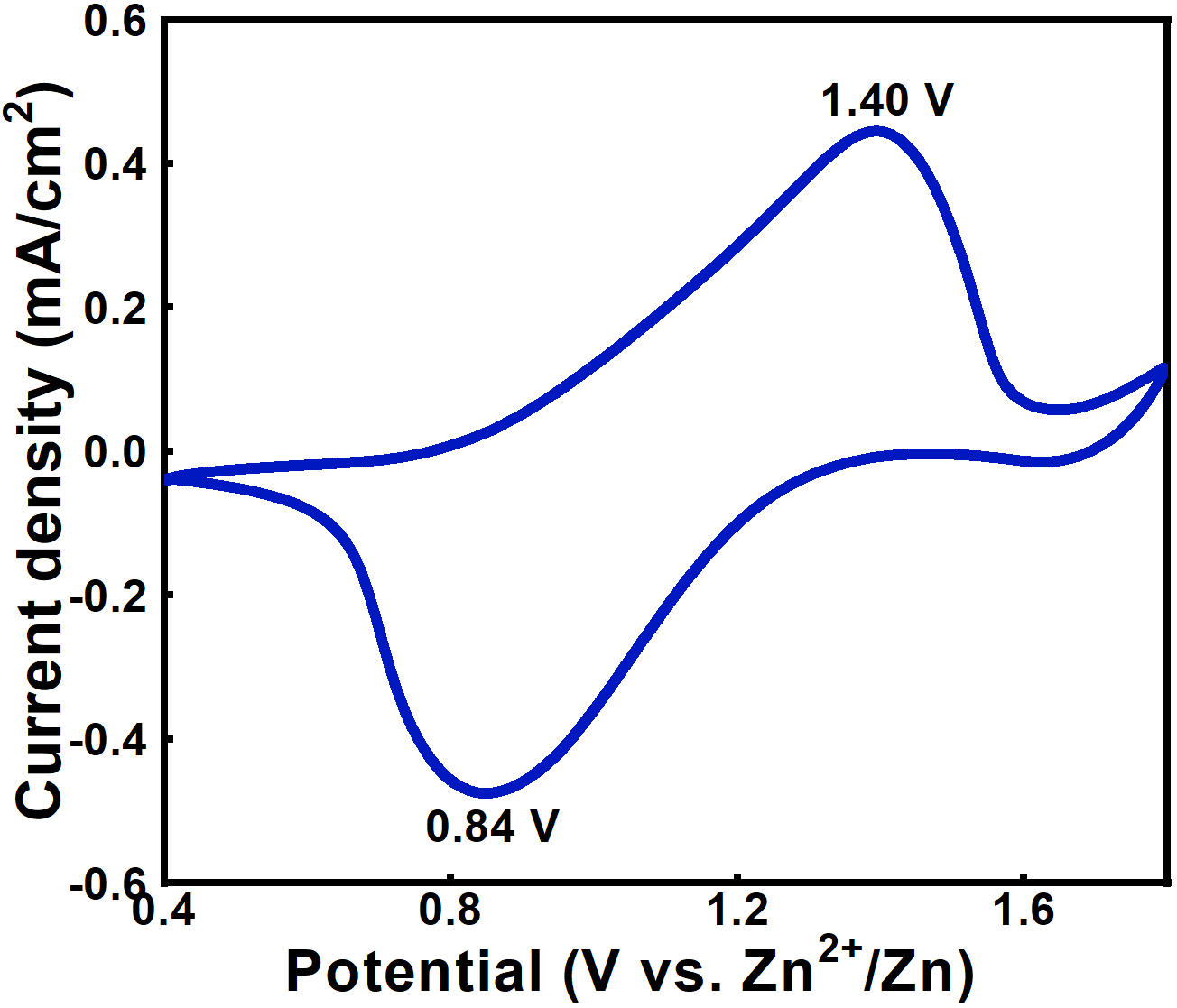


**Figure S14.** Cyclic voltammogram of the Zn-PB ZECD in electrolyte containing 1 M ZnSO_4_ at a scan rate of 50 mV s^-1^ over a voltage range of 0.4-1.8 V.


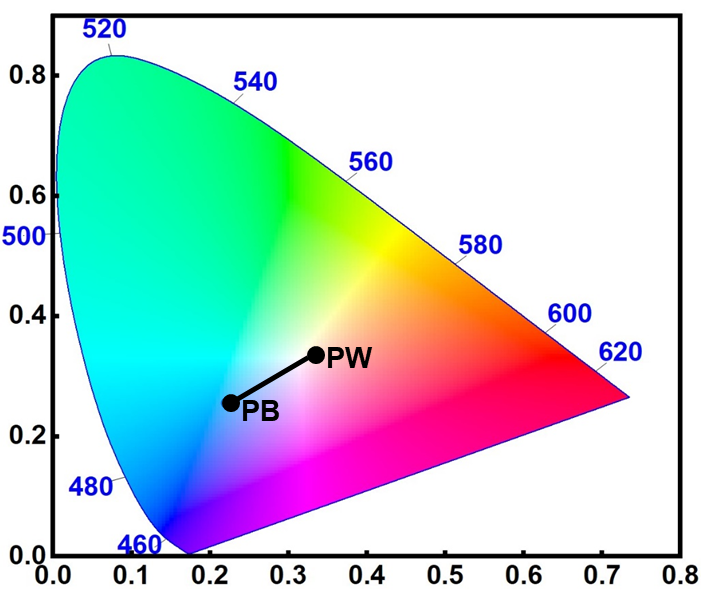


**Figure S15**. Single-layer Zn-PB devices demonstrate one-dimensional linear color transitions. Conventional single-material-based devices (Zn-PB devices) exhibited linear color transitions within a one-dimensional color space, restricting their achievable color gamut.


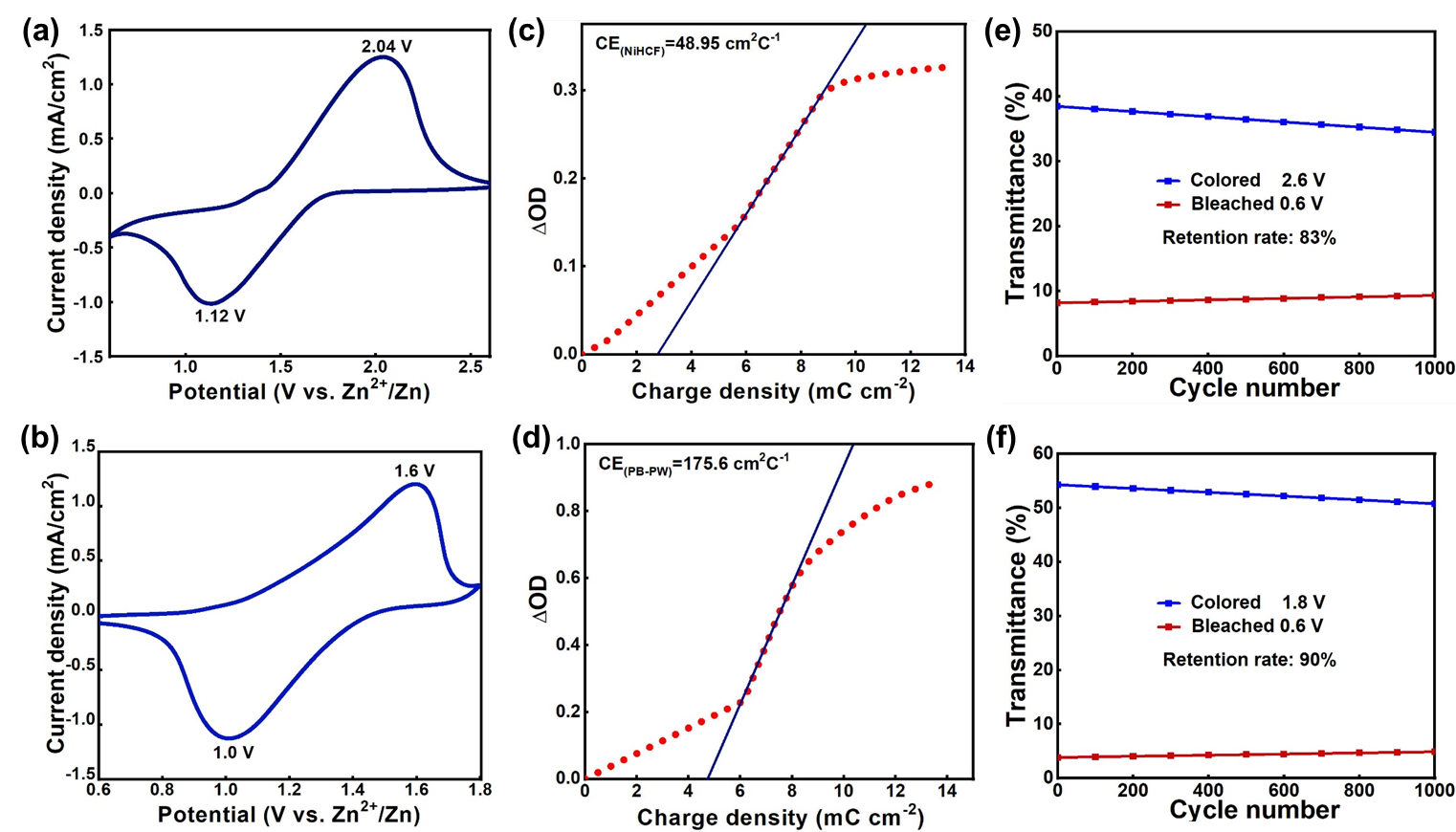


**Figure S16**. Electrochromic performance of the NiHCF-Zn-PB ZECD prototype device. Cyclic voltammogram of (a) the Zn-NiHCF and (b) the Zn-PB in the NiHCF-Zn-PB ZECD at a scan rate of 50 mV s^-1^. Coloration efficiency of the (c) the Zn-NiHCF and (d) the Zn-PB in the NiHCF-Zn-PB ZECD. (e) Zn-NiHCF and (f) Zn-PB device retain 83% and 90% of its initial optical contrast after 1000 cycles, respectively.


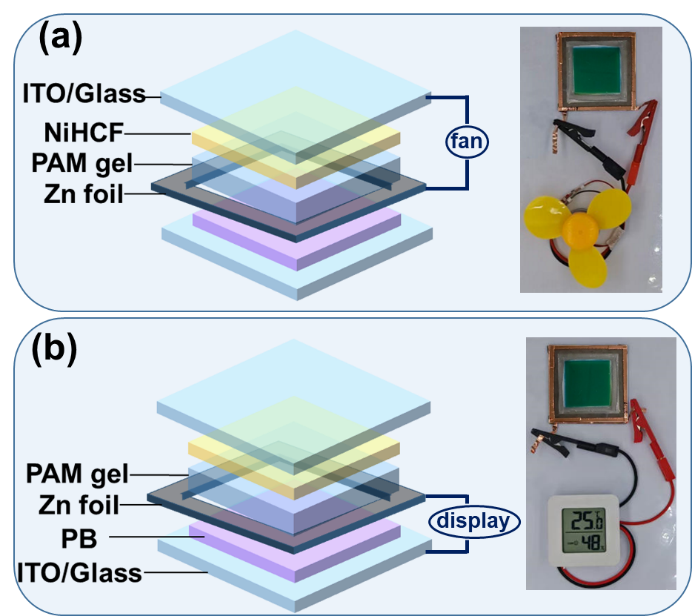


**Figure S17**. Internal potential differences of the NiHCF-Zn-PB can power external minor appliances. (a) The potential gradient of NiHCF and Zn foil can power a fan. (b) The potential gradient of PB and Zn foil can power a temperature-humidity display. The built-in voltage within the rocking-chair NiHCF-Zn-PB system can generate sufficient voltage to power small electronic devices. The OCP between Zn foil and NiHCF/PB can power a small fan and a temperature-humidity display, respectively.

**
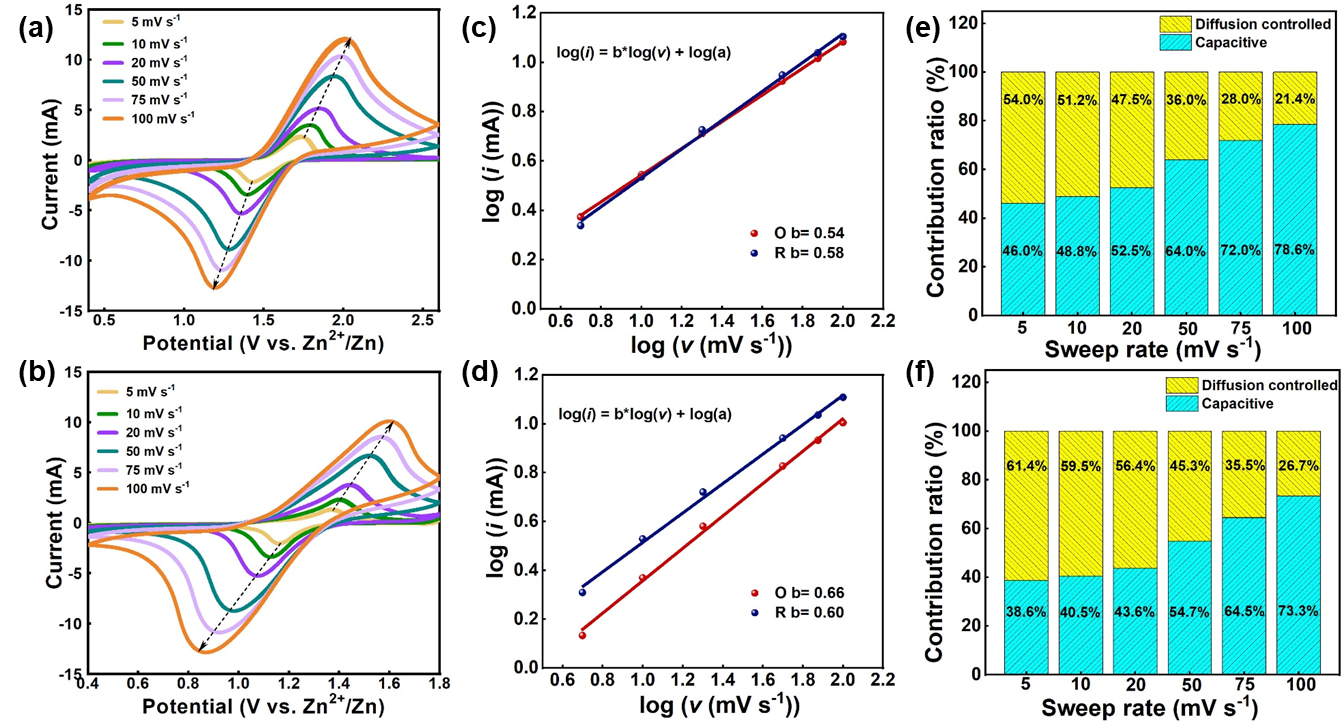
**

**Figure S18**. CV curves recorded at various scan rates of (a) Zn-NiHCF and (b) Zn-PB devices and the linear fittings of log(*i*) *vs.* log(*v*) for (c) Zn-NiHCF and (d) Zn-PB devices at cathodic and anodic peaks. Capacity contributions of battery-type and capacitor-type at different scan rates for (e) Zn-NiHCF and (f) Zn-PB devices.

An electrochemical kinetic method was employed to investigate the mechanism underlying rate capability. Initially, CV curves were collected at scan rates ranging from 5 to 100 mV s^-1^ (Figure S18a, b). According to the function relationship described by the equations: *i* = a*v*^b^ or log(*i*) = b*log(*v*) + log(a)^[3-4]^, linear fitting of log(*i*) *vs.* log(*v*) was performed at both cathodic and anodic peaks, as shown in Figure S18c, d. For the Zn-NiHCF system, the calculated *b*-values of 0.58 and 0.54 at the cathodic and anodic peaks, respectively, imply the dominance of diffusion-controlled battery behavior (Figure S18c). Similarly, for the Zn-PB system, the *b*-values of 0.60 and 0.66 at the cathodic and anodic peaks, respectively, also indicate the dominance of diffusion-controlled battery behavior (Figure S18d).

The contribution of battery-type (diffusion-controlled) and capacitor-type (surface-controlled) behaviors to the capacity was quantified using the equation: *i* = k_1_*v* + k_2_*v*^1/2^, where k_1_v and k_2_*v*^1/2^ stand for the contributions of capacitor-type and battery-type behavior, respectively (Figure S18e-f)^[3-4]^. When the scan rate was increased from 5 to 100  mV s^–1^, the diffusion-controlled contribution in the Zn-NiHCF system decreased from 54.0% to 21.4%, while the capacitive contribution rose from 46.0% to 78.6% (Figure S18e). Similarly, for the Zn-PB system, as the scan rate increased, the diffusion-controlled contribution declined from 61.4% to 26.7%, with a corresponding increase in capacitive contribution from 38.6% to 73.3% (Figure S18f). These results demonstrate a pronounced enhancement of capacitive behavior at elevated scan rates.


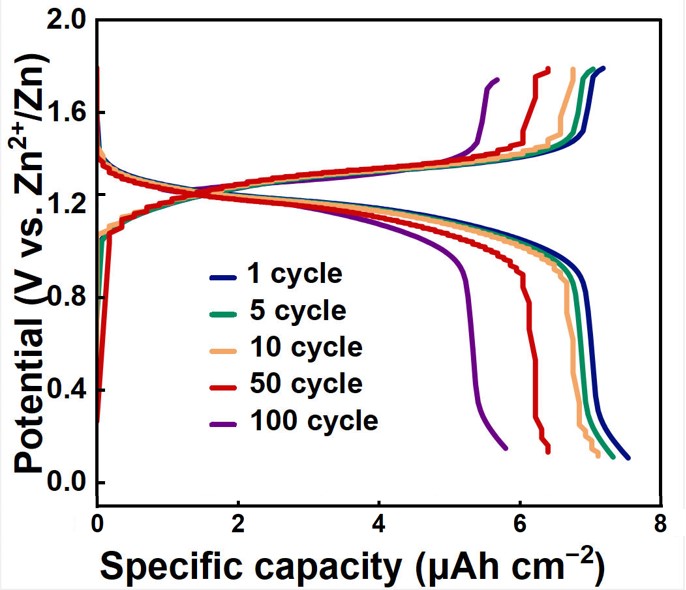


**Figure S19**. Cycling performance of the Zn-PB device employing a PAM hydrogel electrolyte, showing the galvanostatic charge-discharge (GCD) profiles from the 1st, 5th, 10th, 50th, and 100th cycles at current densities of 0.1 mA cm^−2^ for discharging and 0.2 mA cm^−2^ for charging.


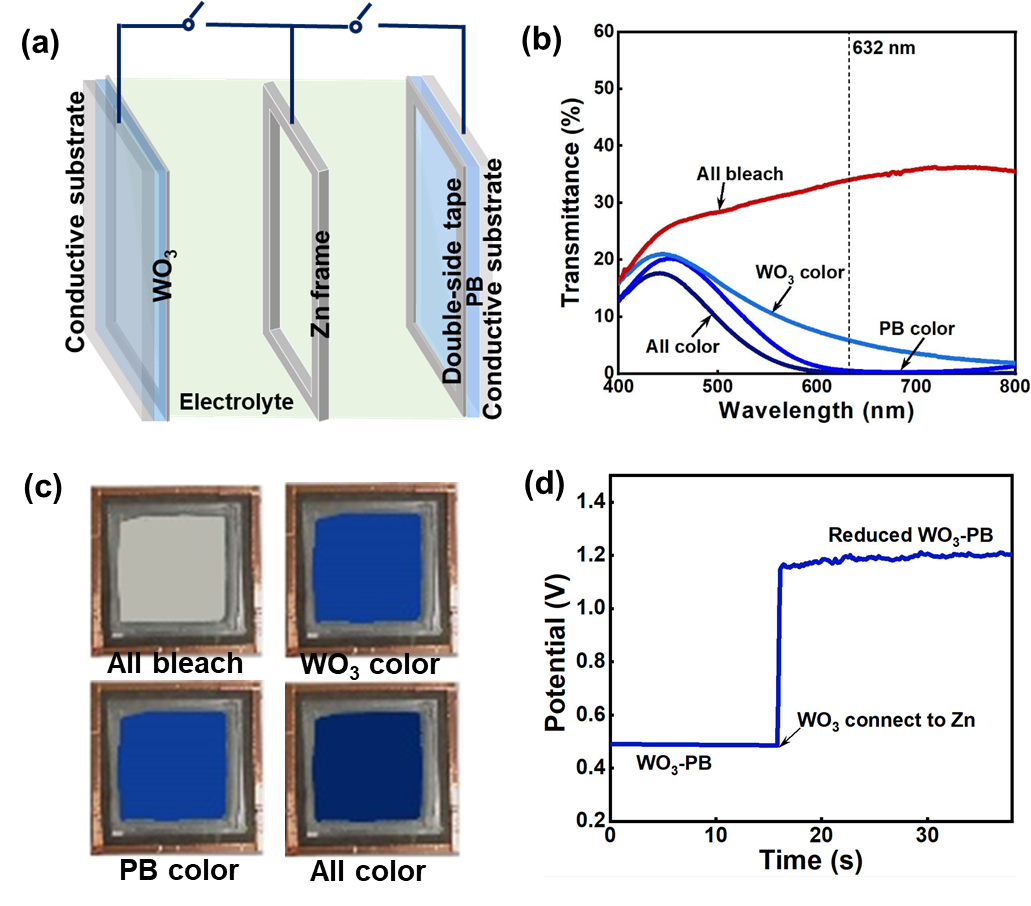


**Figure S20**. Color-switching performance of the PB-Zn-WO_3_ ZECD prototype device. (a) Schematic diagram of the device configuration. (b) Optical transmittance spectra and (c) corresponding digital photographs of the device in the four states: all colored (deep blue), all bleached (transparent), PB color (blue), and WO_3_ color (blue). (d) In-situ electrode potential measurements were conducted for PB, WO_3_ and Zn. The initial potential difference between PB (blue) and WO_3_ (transparent) was approximately 0.5 V. Upon connecting WO_3_ with Zn, the potential gradient between them drove the spontaneous reduction of WO_3_, resulting in a visible color change from transparent to blue. Subsequently, the open-circuit potential between the reduced WO_3_ (blue) and PB increased sharply from ~0.5 V to nearly 1.2 V within 0.1 s. This voltage was sufficient to power an external LED, during which PB underwent a color transition from blue to transparent.


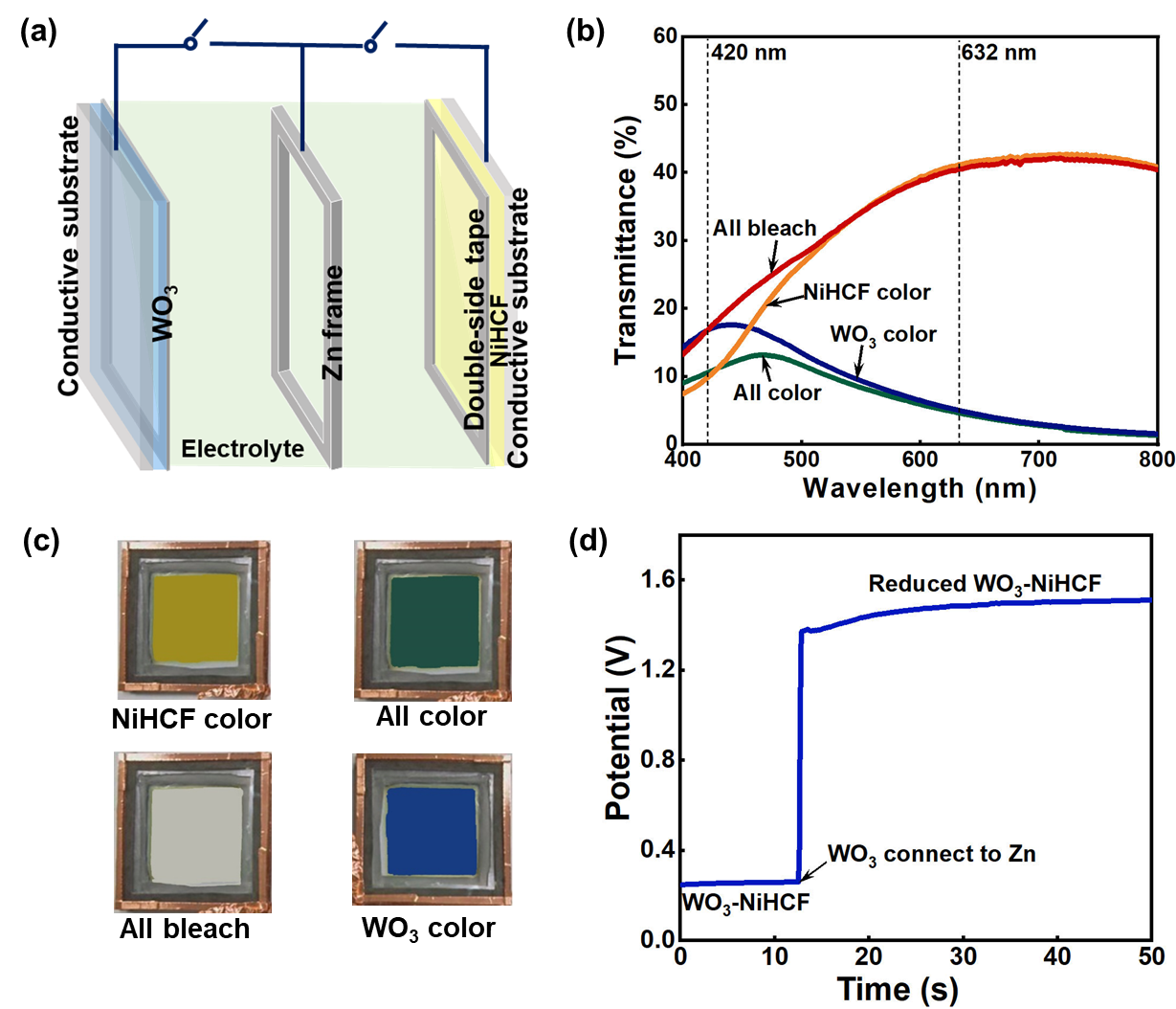


**Figure S21**. Color-switching performance of the NiHCF-Zn-WO_3_ ZECD prototype device. (a) Schematic diagram of the device configuration. (b) Optical transmittance spectra and (c) corresponding digital photographs of the device in the four states: all colored (green), all bleached (transparent), NiHCF color (yellow), and WO_3_ color (blue). (d) In-situ electrode potential measurements were conducted for NiHCF, WO_3_ and Zn. The initial potential difference between NiHCF (yellow) and WO₃ (transparent) was approximately 0.25 V. Upon connecting WO_3_ with Zn, the potential gradient between them drove the spontaneous reduction of WO_3_, resulting in a visible color change from transparent to blue. Subsequently, the open-circuit potential between the reduced WO_3_ (blue) and NiHCF increased sharply from ~0.25 V to nearly 1.5 V. This voltage was sufficient to power an external LED, during which NiHCF underwent a color transition from yellow to transparent.


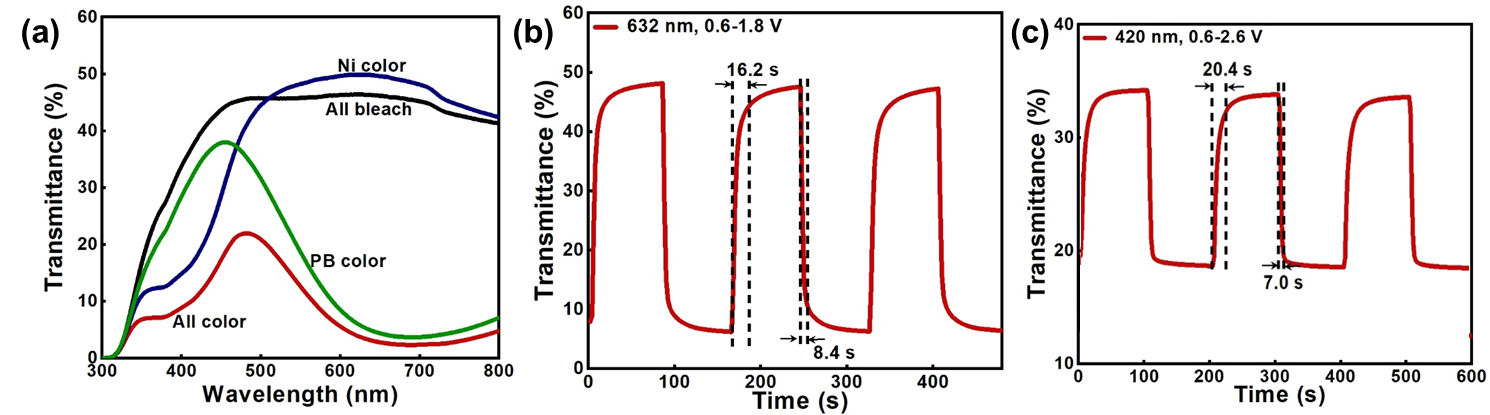


**Figure S22**. Electrochromic performance of the flexible PB-Zn-NiHCF ZECD device. (a) Optical transmittance spectra of the flexible device in different states: all colored (green), all bleached (transparent), PB color (blue), and NiHCF color (yellow). Real-time transmittance changes measured at (b) 632 nm in the 0.6-1.8 V window and (c) 420 nm in the 0.6-2.6 V window.


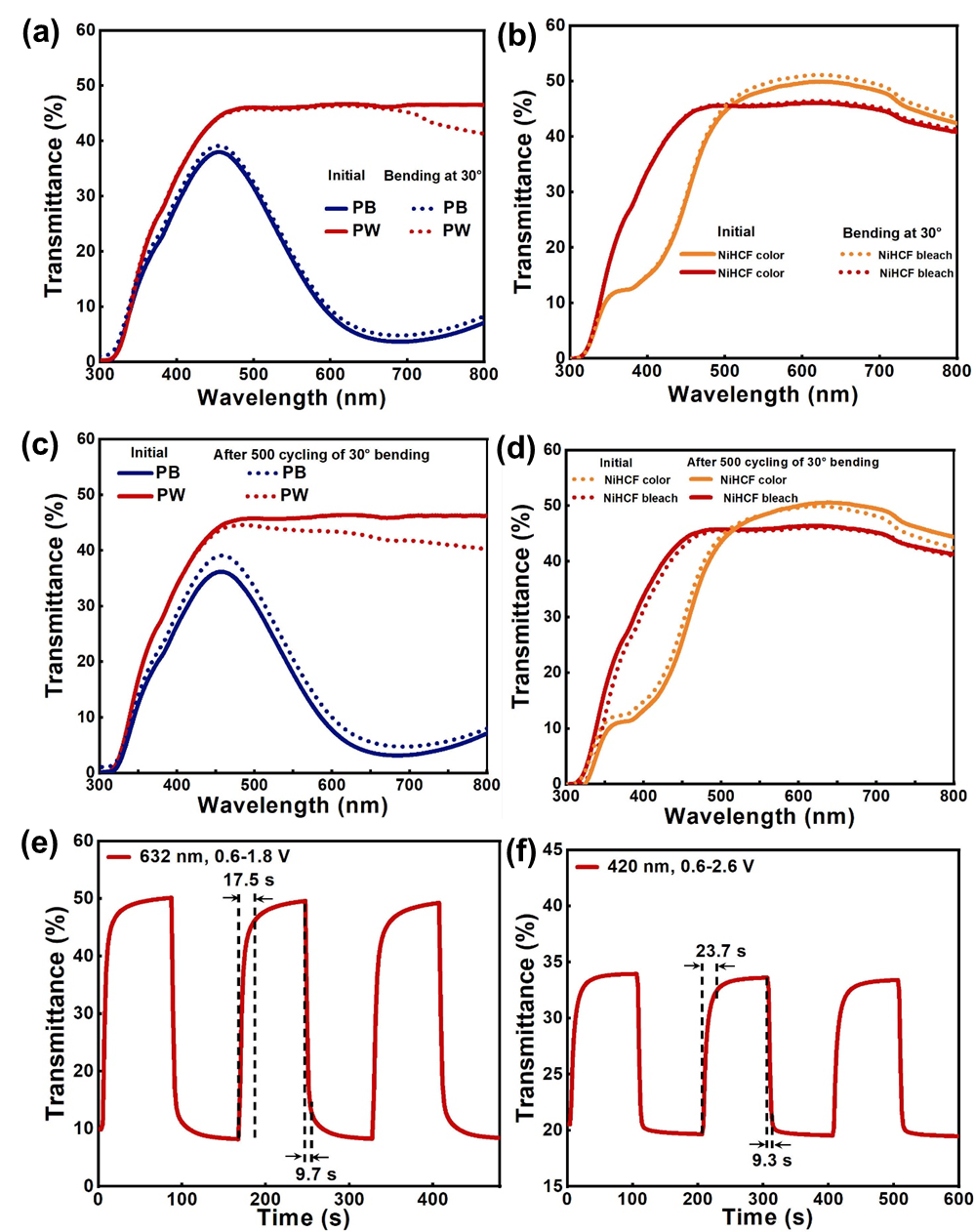


**Figure S23**. Mechanical deformation stability of the flexible PB-Zn-NiHCF ZECD device. (a-b) Optical transmittance spectra of the flexible device while undergoing 30° inward bending. (c-f) Electrochromic performance of the flexible PB-Zn-NiHCF ZECD prototype device after 500 cycles of 30° inward bending. (c-d) Optical transmittance spectra of the flexible PB-Zn-NiHCF device. Real-time transmittance changes of (e) PB and (f) NiHCF measured at 632 nm in the 0.6 V-1.8 V and 0.6-2.6 V windows, respectively. After 500 bending cycles at 30°, the PB-Zn-NiHCF ZECD retained 87% and 98% of the optical modulation performance of the Zn-PB and Zn-NiHCF components, respectively. Notably, the response time remained nearly unchanged, highlighting the device’s excellent mechanical flexibility and operational stability.


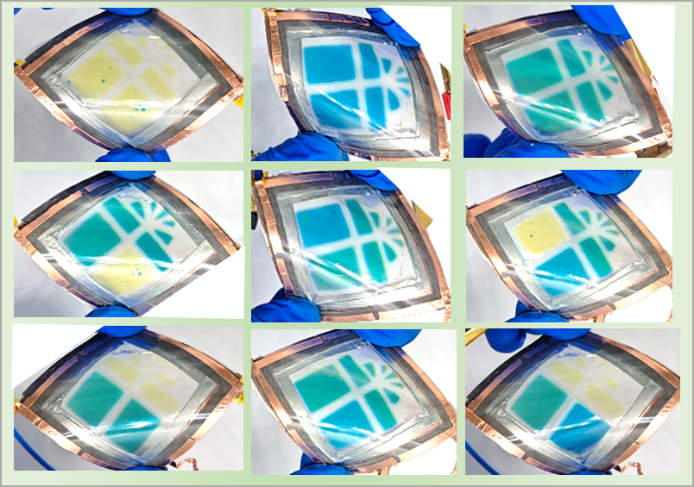


**Figure S24**. Digital photographs of the multi-region patterned multicolor electrochromic system. The multicolor electrochromic system enabled precise, localized color modulation across distinct regions of the device, allowing reversible transitions from transparent to various color states, such as yellow, blue, and green. These transitions could be activated independently or concurrently in designated sections.

**
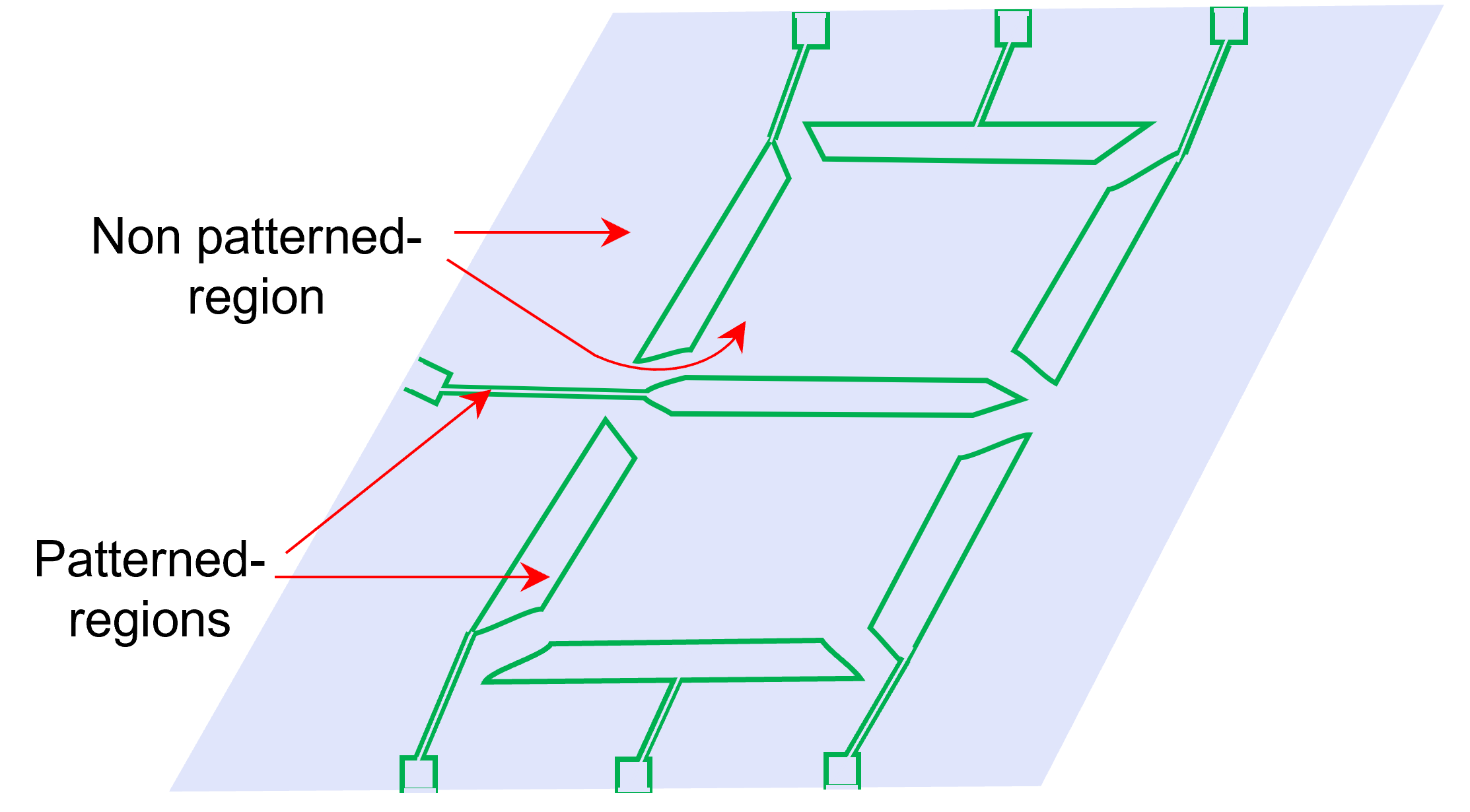
**

**Figure S25**. Details of the patterned “8” that was laser-etched onto an ITO/glass substrate. Each segment formed an independently addressable electrochromic unit (with the channeled connection for wiring) that was one of the seven patterned regions, while all of the remaining areas (other than the 7 segments) were actually one continuous non-patterned region, which was connected to an extra wire.


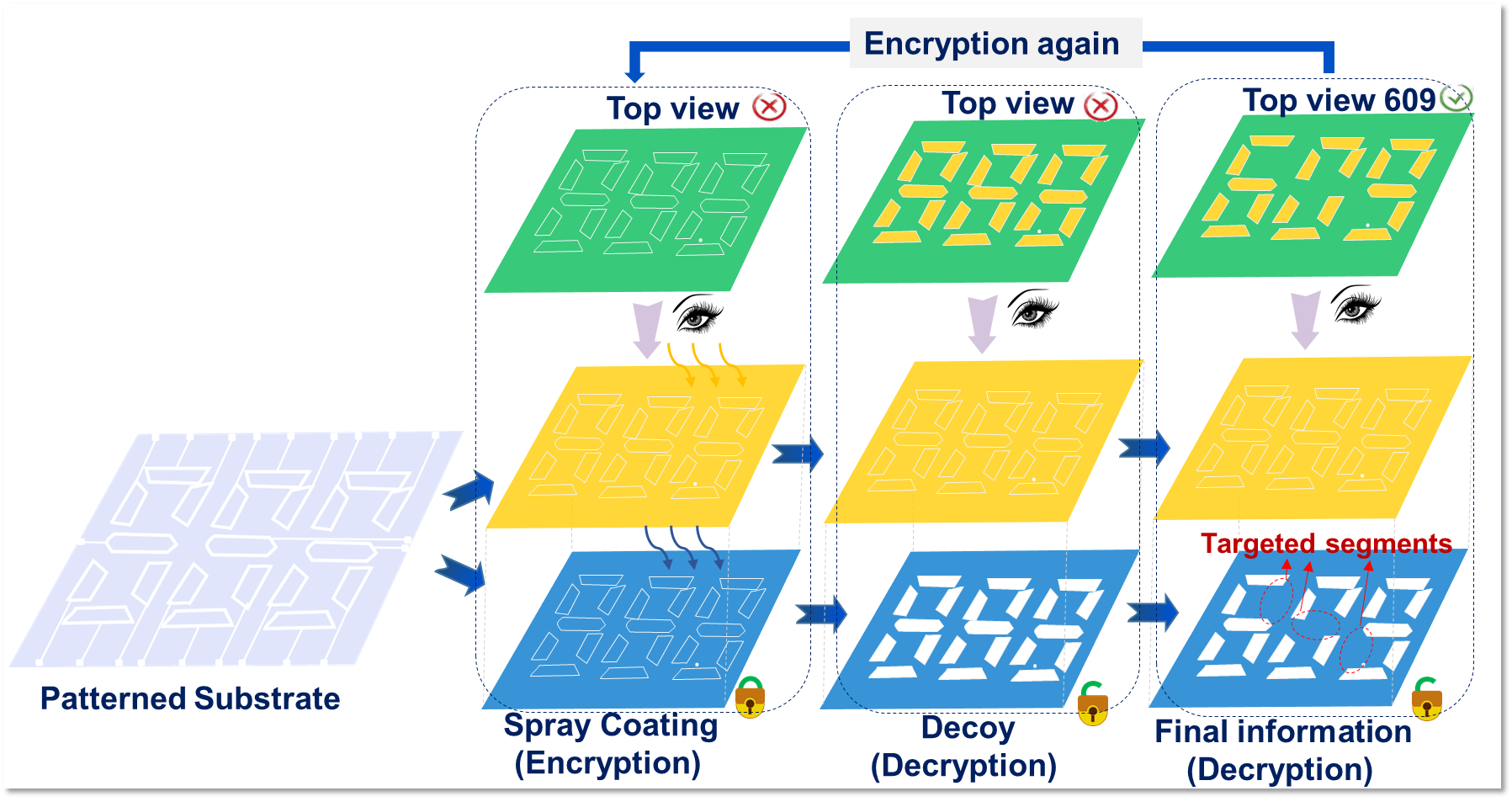


**Figure S26**. Schematic illustration of encryption and decryption of an 888 code based on PB-Zn-NiHCF ZECDs. A laser-etched 888 was created on an ITO/glass substrate (Patterned substrate). Then, NiHCF and PB materials were evenly sprayed over the entire surface, embedding the hidden yellow/blue 888 into the device (Encryption). The display appeared uniformly green due to the color overlay effect. Through sequential voltage application strategy, the second layer of hidden information, the actual information 609 in yellow color with green background is revealed (Final information). The corresponding real photographs of the PB-Zn-NiHCF device was shown in Figure 4c, i-ii.


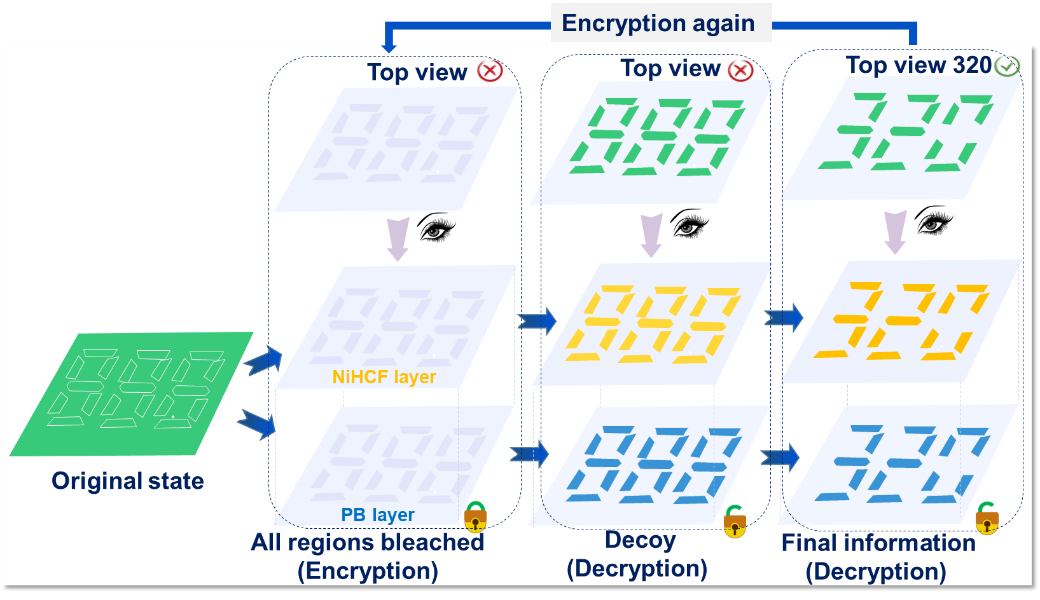


**Figure S27**. Schematic illustration of encryption and decryption of an 888 code based on PB-Zn-NiHCF ZECDs. Through sequential voltage application strategy and color overlay effect, the hidden information, the actual information 320 in green color with transparent background was revealed (Final information). The corresponding real photographs of the PB-Zn-NiHCF device was shown in Figure 4d, i-ii.


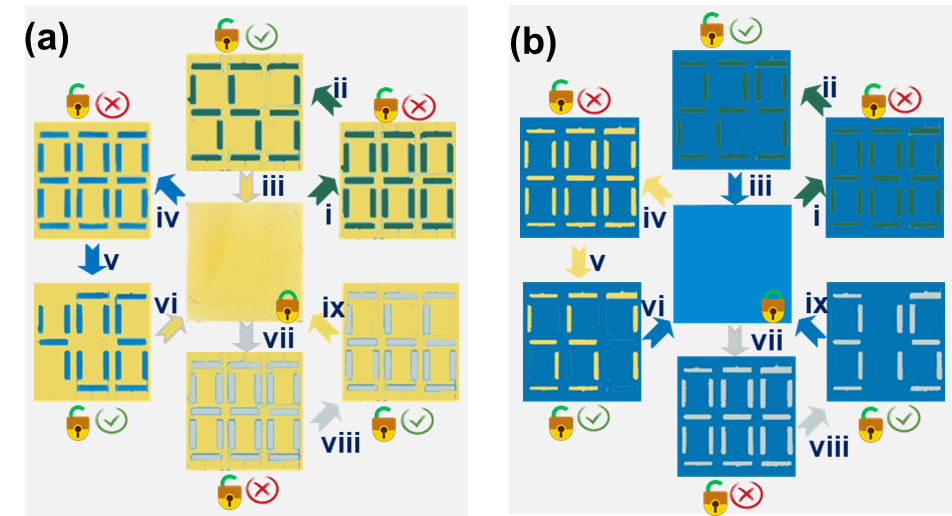


**Figure S28**. Real photographs of the PB-Zn-NiHCF device. Multi-level information display process utilizes colored (a) NiHCF / (b) PB layers on (a) yellow / (b) blue backgrounds, enabling selective visualization of numerals in green, (a) blue / (b) yellow, or transparent states. The system enabled dynamic multicolor numeral displays across various backgrounds by selectively bleaching and coloring distinct electrode regions. The ZECD could render green (553), blue (406), and colorless (666) numerals on a yellow background (Figure S28a), and similarly display green (229), yellow (557), and colorless (315) numerals against a blue background (Figure S28b). This versatility underscores the platform’s spatially controlled electrochemical tuning capability.


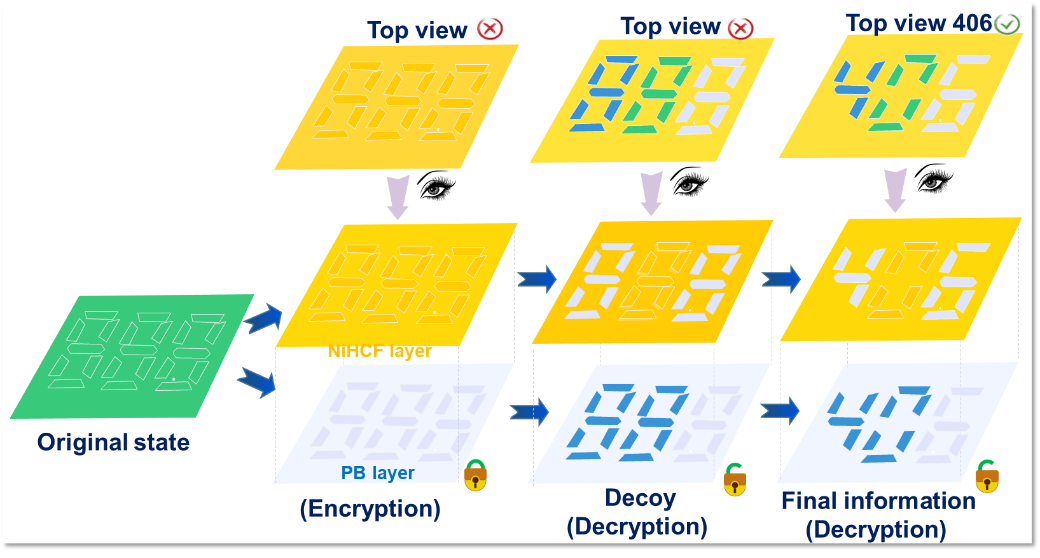


**Figure S29**. Schematic illustration of dynamic electrochromic decryption system executed stepwise decryption through multistep color switching. The device could simultaneously display 888 in three distinct colors, blue, green, and transparent (against a yellow background; decoy (decryption)), and was capable of selectively decrypting it into 406 while maintaining the same color configuration (final information). The corresponding real photographs of the PB-Zn-NiHCF device was shown in Figure 4e, i-ii.


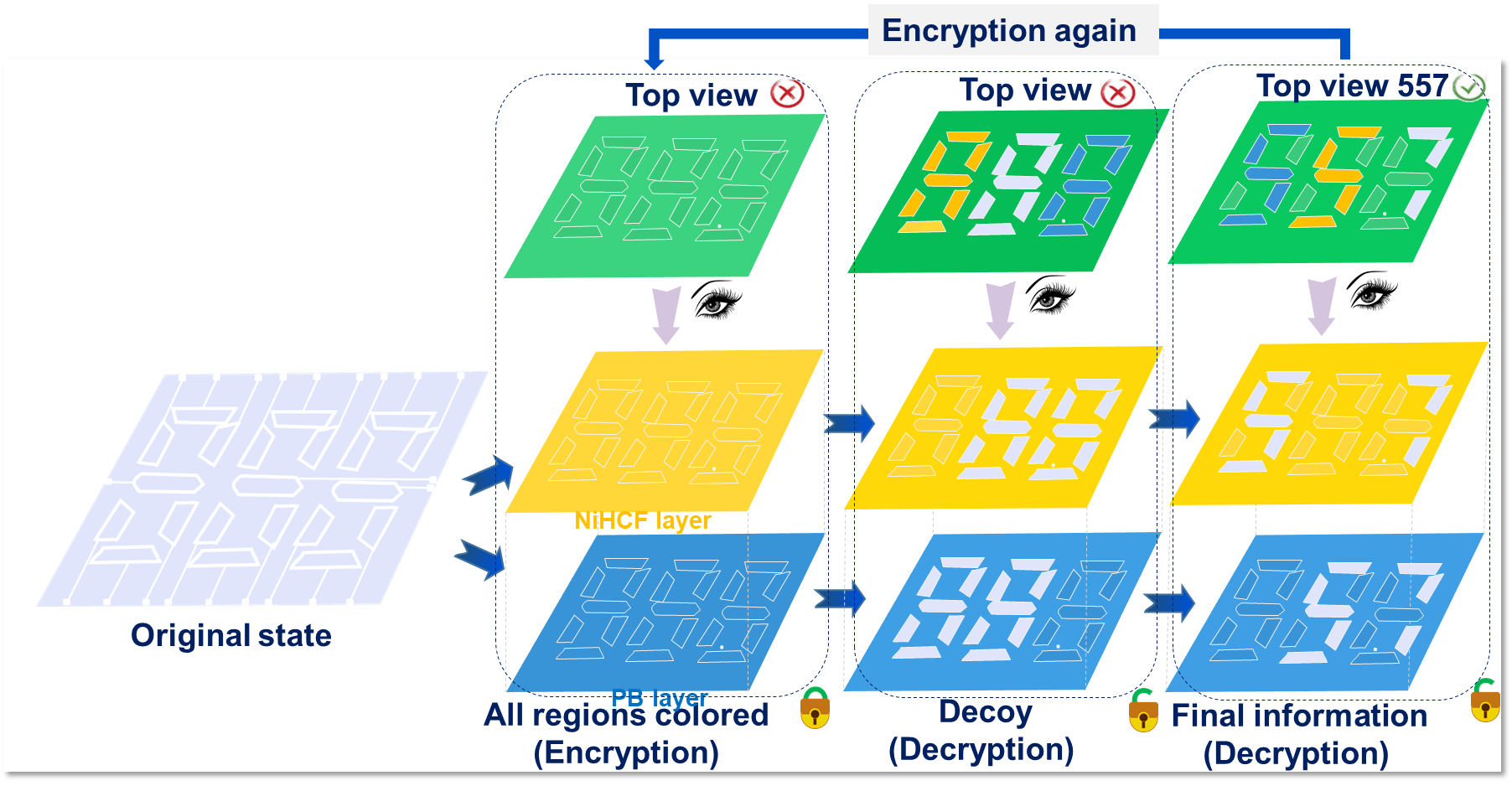


**Figure S30**. Schematic illustration of dynamic electrochromic decryption system executed stepwise decryption through multistep color switching. The device could simultaneously display 888 in three distinct colors, yellow, transparent, and blue (against a green background; decoy (decryption)), and was capable of selectively decrypting it into 557 while exhibiting different color configuration (blue, yellow, and transparent; final information). The corresponding real photographs of the PB-Zn-NiHCF device were shown in Figure 4e, iv-v.


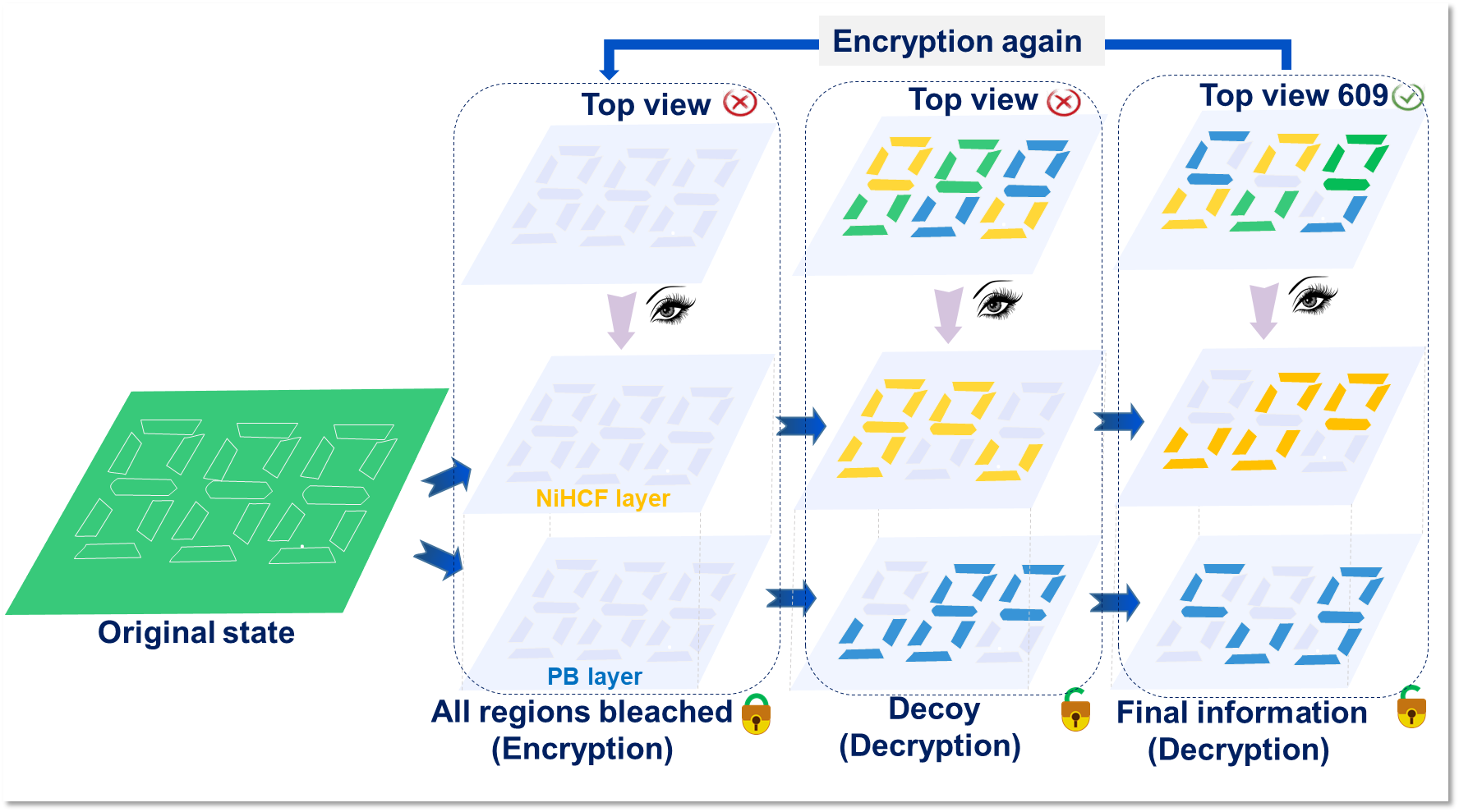


**Figure S31**. Schematic illustration of dynamic electrochromic decryption system executed stepwise decryption through multistep color switching. The device could simultaneously display 888 in different color segments, yellow-green, green-blue, and blue-yellow (against a transparent background; decoy (decryption)), and was capable of selectively decrypting it into 609 while exhibiting different color configurations (blue-yellow, yellow-green, and green-blue; final information). The corresponding real photographs of the PB-Zn-NiHCF device were shown in Figure 4e, xi-xii.


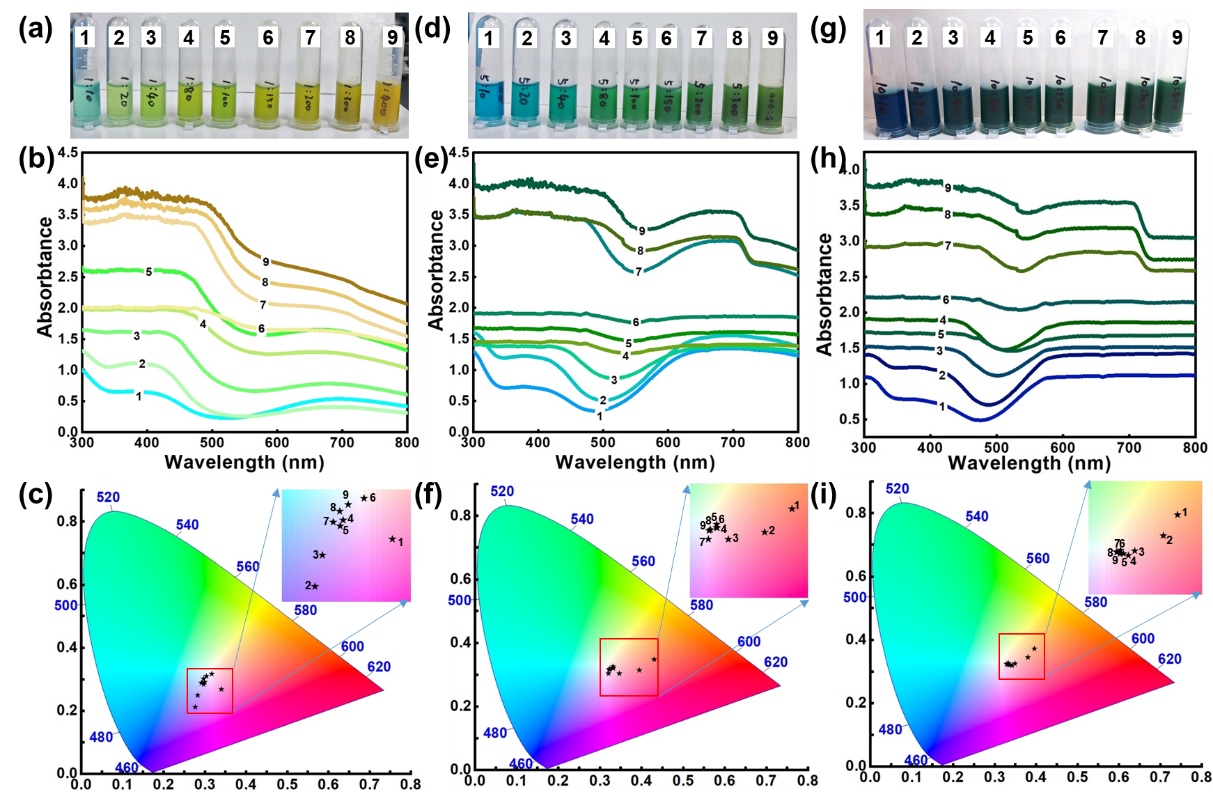


**Figure S32**. Multicolor solutions with varying PB/NiHCF ratios for information security applications. Solutions with PB:NiHCF ratios from 1:10 to 1:400 show (a) photographs, (b) optical absorption spectra, and (c) CIE coordinates (numbers correspond to colors in a) across light blue-green-yellow states. Solutions with PB:NiHCF ratios from 5:10 to 5:400 displaying (d) photographs, (e) absorption spectra, and (f) CIE coordinates for blue/blue-green/green states. 10:10-10:400 ratio films exhibit (g) photographs, (h) absorption spectra, and (i) CIE coordinates across dark blue/blue-green/green states. All ratios demonstrate nine distinct color states, with numbered labels corresponding to colors in each panel. We established a nanoparticle-blending strategy incorporating PB and NiHCF, enabling fine-tuned color modulation by adjusting their compositional ratio. This approach preserved high color purity while producing a wide array of vibrant hues.

**Table S1.** Comparison of cyclic voltammetry, coloration speed, coloration efficiency, optical contrast, and cycling stability of individual Zn-PB and Zn-NiHCF devices with those of the integrated dual-layer device.

| **Device** | **Cyclic voltammogram**  **(CV, V)** | **Optical modulation (ΔT)** | **Response time** | **Coloration efficiency**  **(CE, cm^2^ C^-1^)** | **Cycling stability retention rate**  **(after 1000 cycles)** |
| --- | --- | --- | --- | --- | --- |
| Zn-NiHCF | 1.1 / 2.0 | 32.6%  at 420 nm | T_b_=18.4 s  T_c_=9.3 s  At 420 nm | 42.5 | 85% |
| Zn-PB | 0.9 / 1.5 | 63.0%  at 632 nm | T_b_=16.7 s  T_c_=12.9 s  at 632 nm | 167.2 | 92% |
| Zn-NiHCF  (PB-Zn-NiHCF) | 1.1 / 2.0 | 30.2%  at 420 nm | T_b_=25.6 s  T_c_=8.0 s  at 420 nm | 48.9 | 83% |
| Zn-PB  (PB-Zn-NiHCF) | 1.0 / 1.6 | 50.4%  at 632 nm | Tb=15.4 s  Tc=9.2 s  at 632 nm | 175.6 | 90% |

**Table S2.** Comparison of encryption efficiency, security level, and dynamic tenability, switchable color, cycling stability, and cycling stability of various previously reported electrochromic encryption platforms.

| **Encryption**  **efficiency** | **Security level** | **Dynamic tenability** | **Flexibility** | **Types of Colors** | **Cycling stability** | **External voltage (V)** | **Ref.** |
| --- | --- | --- | --- | --- | --- | --- | --- |
| **8.0 s - 25.6 s** | **Multi modes** | **Dynamic;**  **on demand** | **Flexible state** | **Multicolor** | **1000** | **0.6 ~ 2.6 (or self-power)** | **This work** |
| 2.56 s | Dual modes | Pre-designed; static state | Flexible state | Transparent to green | 50 | 0.8 ~ 2.0 | [5] |
| 12 s - 100 s | Dual modes | Pre-designed; static state | Rigid state | Multicolor | 35 | 5 ~ 22 | [6] |
| 2.6 s - 2.8 s | Dual modes | Pre-designed | Rigid state | Multicolor | 7200 | -4 ~ 4 | [7] |
| 1 μs | Single mode | Static state | Rigid state | Single | NA | -5 ~ 4 | [8] |
| 760 ms | Dual modes | Dynamic modulation | Rigid state | Multicolor | 50 | -0.2 ~ 0.8 | [9] |
| NA | Multi modes | Static state | Rigid state | Multicolor | NA | 8 | [10] |
| 5 min | Single mode | Static state | Rigid state | Green to yellow | NA | 5 ~ 10 | [11] |

**References**

[1] L.-Y. Hsiao, F.-Y. Kuo, C.-H. Wu, Y.-C. Huang, Y.-C. Wang, R.-J. Jeng, K.-C. Ho, *Chem. Eng. J.* **2021**, *420*, 129821.

[2] F. Zhao, J. Zhao, Y. Zhang, X. Wang, W. Wang, *J. Mater. Chem. C* **2021**, *9*, 7958.

[3] J. Sun, Z. Zhang, G. Lian, Y. Li, L. Jing, M. Zhao, D. Cui, Q. Wang, H. Yu, C.-P. Wong, *ACS Nano* **2022**, *16*, 12425.

[4] J. Sun, G. Lian, L. Jing, D. Wu, D. Cui, Q. Wang, H. Yu, H. Zhang, C.-P. Wong, *Nano Research* **2022**, *15*, 4108.

[5] D. Yu, E. Jang, Y. Wi, J. Hyeong, K. M. Lee, N. P. Godman, M. E. McConney, L. De Sio, S. I. Lim, K. U. Jeong, *Adv. Funct. Mater.* **2025**, *35*, 2420062.

[6] D. Yang, Y. Lu, X. Chen, X. Wang, H. Cao, Z. Yang, W. He, Z. Miao, Y. Luan, X. Du, D. Wang, *Chem. Eng. J.* **2025**, *513*, 162833.

[7] T. Zhang, X. Mu, Y. Li, S. Cong, S. Zheng, R. Huang, F. Geng, Z. Zhao, *Adv. Mater.* **2024**, *36*, 2402670.

[8] K. Wang, W. Li, Y. Liao, J. Li, R. Chen, Q. Chen, B. Shi, D. H. Kim, J. H. Park, Y. Zhang, X. Zhou, C. Wu, Z. Liu, T. Guo, T. W. Kim, *Adv. Mater.* **2024**, *36*, e2306065.

[9] J. H. Ko, J. E. Yeo, H. E. Jeong, D. E. Yoo, D. W. Lee, Y. W. Oh, S. Jung, I. S. Kang, H. H. Jeong, Y. M. Song, *Nanophotonics* **2024**, *13*, 1119.

[10] Q. Zhao, W. Xu, H. Sun, J. Yang, K. Y. Zhang, S. Liu, Y. Ma, W. Huang, *Adv. Opt. Mater.* **2016**, *4*, 1167.

[11] W. Lin, Q. Zhao, H. Sun, K. Y. Zhang, H. Yang, Q. Yu, X. Zhou, S. Guo, S. Liu, W. Huang, *Adv. Opt. Mater.* **2014**, *3*, 368.
